# RNASET2 Deficiency Induces Hepatocellular Carcinoma Metastasis through Cholesterol‐Triggered MET Activation

**DOI:** 10.1002/advs.202411888

**Published:** 2025-02-04

**Authors:** Yanquan Xu, Yu Chen, Jiangang Zhang, Jingchun Wang, Shuai Yang, Huakan Zhao, Lei Wu, Juan Lei, Yu Zhou, Jin Peng, Lu Jiang, Qian Chen, Rong Xin, Jianyun Zhou, Yongsheng Li

**Affiliations:** ^1^ Clinical Medicine Research Center Xinqiao Hospital Army Medical University Chongqing 400037 China; ^2^ Department of Medical Oncology Chongqing University Cancer Hospital Chongqing 400030 China; ^3^ Department of Gastroenterology Xinqiao Hospital Army Medical University Chongqing 400037 China; ^4^ Department of Pathology The 958th Hospital Southwest Hospital Army Medical University Chongqing 400037 China

**Keywords:** cholesterol, hepatocellular carcinoma, metastasis, MET, ribonuclease

## Abstract

Metastasis remains a significant challenge in the treatment of hepatocellular carcinoma (HCC). The role of ribonuclease T2 (RNASET2) in HCC is still uncertain, although it has been reported to have contradictory effects on some cancers. Here, it is demonstrated that RNASET2 knockout leads to significant accumulation of cholesterol, which in turn promotes MET‐mediated HCC metastasis. Mechanistically, the absence of RNASET2 hinders the degradation of RNA into uridine, thereby reducing the conversion to UTP. This reduction restrains glucuronate metabolism and the expression of the related enzyme UDP‐glucuronosyltransferase (UGT)1A1, ultimately resulting in the accumulation of cholesterol due to decreased formation of glucuronidated‐bile acids. The administration of cholesterol induces the migration and invasion of HCC cells through MET (mesenchymal‐epithelial transition factor) activation. However, the deficiency of RNASET2‐induced HCC metastasis can be reversed by blocking MET with shRNA or savolitinib. The study identifies RNASET2 as a key regulator that coordinates RNA, glucuronate, and cholesterol metabolism. Its deficiency drives HCC metastasis through cholesterol‐triggered MET activation. These findings highlight the potential of targeting RNASET2 and MET in improving the prognosis of HCC.

## Graphical abstract

This study elucidates the role of RNASET2 in inhibiting the metastasis of HCC. RNASET2 preferentially cleaves RNA into uridine, which contributes to the salvage synthesis of UTP and the glucuronidation of bile acids, thereby maintaining a balance in cholesterol metabolism. This process is followed by the degradation of MET in the lysosome. In HCC cell lines, the deficiency of RNASET2 leads to impaired glucuronate metabolism and an accumulation of cholesterol, which activates the MET signaling pathway and promotes HCC metastasis.

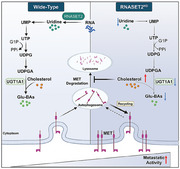

## 1 Introduction

Liver cancer, the seventh most diagnosed cancer globally, is also the leading cause of cancer‐related death.^[^ ^1^ ^]^ Hepatocellular carcinoma (HCC) makes up over 80% of liver cancer cases and has a poor clinical prognosis due to its high metastatic rate.^[^ ^2^ ^]^ Tumor cells undergo metabolic reprogramming and epithelial mesenchymal transition (EMT) to initiate invasion and metastasis, a process regulated by various intra‐ and extra‐cellular signaling pathways.^[^ ^3^, ^4^, ^5^, ^6^ ^]^ Among which, receptor tyrosine kinases (RTKs) that respond to growth factors have been found to play crucial roles in malignant transformation and cancer metastasis.^[^ ^7^, ^8^, ^9^, ^10^ ^]^ The relationship between metabolic reprogramming and RTKs has been extensively investigated.^[^ ^11^, ^12^, ^13^, ^14^, ^15^ ^]^ Therefore, the identification of RTK suppressors and the clarification of underlying mechanisms hold promise for novel therapeutic interventions in HCC metastasis.

Ribonucleases (RNases) are a type of RNA hydrolases that play a role in regulating both endogenous RNA metabolic homeostasis and the cleavage of exogenous RNA into specific pathogen‐related molecular patterns, which in turn activate inflammatory responses.^[^ ^16^, ^17^, ^18^ ^]^ Humans express a diverse range of RNases, including the endonucleases from the RNase A and RNase T2 families.^[^ ^19^ ^]^ Enzymes from the human RNase A family, such as RNase 5 and RNase 7, have previously been identified as ligands for RTKs to promote the metastasis of solid cancers.^[^ ^20^, ^21^ ^]^ Members of the RNase T2 family possess a highly conserved structure and exert important functions by cleaving RNA. For instance, in Zebrafish, a mutation in RNase T2 leads to neurodegeneration due to the accumulation of non‐degraded RNA in neurons through endolysosomes.^[^ ^22^ ^]^ RNase T2 catalyzes the cleavage of RNA into autophagic uracil, thereby reducing the sensitivity of colorectal cancer (CRC) cells to 5‐fluorouracil (5‐FU).^[^ ^23^ ^]^ The release of uridine from RNA by RNase T2 serves as a ligand for TLR8 activation.^[^ ^17^, ^18^ ^]^ ACTIBIND, a fungal homolog of RNase T2, has been shown to compete with RNase 5 and inhibit melanoma growth and metastasis.^[^ ^24^ ^]^ Interestingly, RNASET2, the only member of the RNase T2 family in humans, has been reported as a regulator in various cancer progressions, including CRC, ovarian cancer, and clear cell renal cell carcinoma.^[^ ^23^, ^25^, ^26^, ^27^ ^]^ These studies suggest that RNASET2 may be involved in the regulation of RTKs‐mediated signal transduction cascades, however, its role in HCC metastasis remains poorly understood.

In this study, we aimed to investigate the role and mechanism of RNASET2 in regulating HCC invasion and metastasis, using clinical subjects and mouse model, thereby providing a potential new therapeutic target for combating HCC metastasis.

## 2 Results

### 2.1 RNASET2 as a Negative Regulator of HCC Invasion and Metastasis

**Figure 1:**
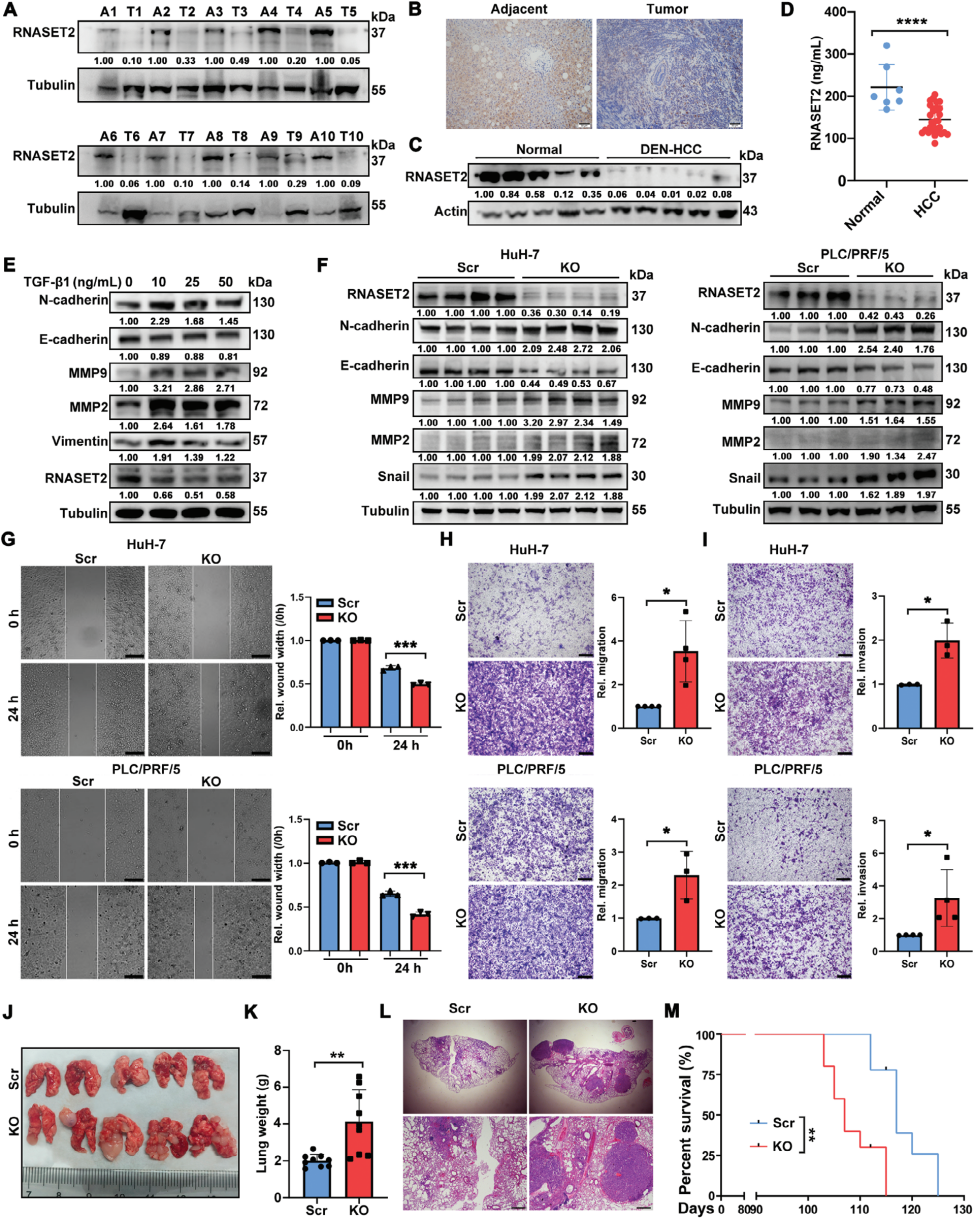
RNASET2 negatively regulates HCC invasion and metastasis: A) Levels of RNASET2 in 10 pairs of HCC tumor tissues (T) and matched adjacent noncancerous tissues (A) were determined by WB (*n* = 10). B) Representative IHC staining images of RNASET2 in tumor tissues and matched adjacent noncancerous tissues of HCC patients (*n* = 12). Scale bars, 50 µm. C) Detection of RNASET2 levels in DEN‐induced mouse HCC tumor tissues and normal mouse liver tissues by WB (*n* = 5). D) ELISA test of soluble RNASET2 levels in plasma samples of HCC patients (*n* = 30) and healthy control group (*n* = 7). E) PLC/PRF/5 cells were treated with the indicated concentrations of TGF‐β1 for 48 h. The expression of RNASET2 and EMT‐related proteins (N‐cadherin, E‐cadherin, MMP9, MMP2, and Vimentin) were measured by WB (*n* = 3). F) Determination of EMT‐related proteins (N‐cadherin, E‐cadherin, MMP9, MMP2, and Snail) in RNASET2^KO^ HuH‐7 (*n* = 4) and PLC/PRF/5 (*n* = 3) monoclonal cell lines utilizing WB. G‐I) Migration and invasion of HuH‐7 and PLC/PRF/5 cells with/without RNASET2 deletion were individually identified by wound healing assay (G), trans‐well migration assay (H), and trans‐well invasion assay (I). The distance of the wound gaps was determined using ImageJ. The wound width was normalized against that at time 0 h. Quantification was shown in the right panel (*n* = 3 to 4). The trans‐well migrated and invaded cells were stained with crystal violet. Bars for wound healing assays: 200 µm, for trans‐well assays: 400 µm. J,K) Images (J) and weight (K) analysis of lung metastatic foci of RNASET2^KO^ HuH‐7 cells (*n* = 9). L) Representative H&E staining of lung metastasis of RNASET2^KO^ HuH‐7 cells compared to scrambled controls in nude mice (*n* = 9). The zoomed images are shown below and the scale bars represented 400 µm. M) Survival of the mice in lung metastasis mouse model injected with RNASET2^KO^ HuH‐7 cells or control cells (*n* = 8). Data are presented as mean ± SD. *p < 0.05, **p <0.01, ***p <0.001, ****p < 0.0001 by two‐tailed unpaired Student's *t*‐test (D,G–I, and K) or log‐rank (Mantel‐Cox) test (M).

We first assessed the expression of RNASET2 in tumor tissues versus matched adjacent non‐tumor tissues in HCC patients and N‐nitrosodiethylamine (DEN)‐induced mouse HCC model using Western blot (WB) and immunohistochemistry (IHC) staining. RNASET2 was decreased in HCC tissues (**Figure** **1**A–C). Similarly, the concentration of RNASET2 in plasma of HCC patients was lower than in the healthy group (Figure 1D). Additionally, the elevated levels of RNASET2 were observed in cell lines with lower invasive and metastatic capabilities (HepG2, PLC/PRF/5, HuH‐7, and BEL‐7404) compared to those with higher metastatic potentials (MHCC97‐H, HCCLM3, and SMMC‐7721) (Figure S1A,B, Supporting Information). Transforming growth factor beta 1 (TGF‐β1) treatment significantly enhanced the expression of mesenchymal markers (N‐cadherin, MMP2, MMP9, Vimentin, and ZEB1), while repressing RNASET2 and the epithelial marker E‐cadherin expression (Figure 1E; Figure S1C, Supporting Information). These findings suggest that a low level of RNASET2 is closely correlated with high HCC malignancy and metastasis.

To investigate the role of RNASET2 in HCC invasion and metastasis, RNASET2 was initially knocked out in highly expressed cell lines, including HepG2, PLC/PRF/5, and HuH‐7. We selected the effective knockout (KO) cell clones for further study (Figure S1D, Supporting Information). As expected, RNASET2^KO^ HCC cells exhibited a higher expression of mesenchymal markers, N‐cadherin, MMP9, MMP2, and Snail1, compared to scrambled (Scr) control HCC cells (Figure 1F; Figure S1E, Supporting Information). Consequently, RNASET2 ablation significantly accelerated the migration and invasion of PLC/PRF/5 and HuH‐7 cells in vitro (Figure 1G–I). Consistently, mice injected with RNASET2^KO^ HCC cells showed a notable increase in the capacity of lung metastasis, and had poorer survival than those in the control group (Figure 1J–M). Similar result was observed in a peritoneal metastasis mouse model inoculated with RNASET2^KO^ HCC cells (Figure S1F, Supporting Information).

To further validate the inhibitory contribution of RNASET2 to the invasion and metastasis of HCC cells, we conducted RNASET2 overexpression in a RNASET2^low^ HCC cell line, MHCC97‐H. The overexpression efficiency was confirmed by qRT‐PCR and WB assays (Figure S1G,H, Supporting Information). RNASET2 overexpression inhibited the EMT, migration, and invasion of MHCC97‐H cells in vitro (Figure S1H‐K, Supporting Information). Furthermore, the exogenous expression of RNASET2 restrained lung metastasis of MHCC97‐H cells and prolonged mouse survival (Figure S1L‐O, Supporting Information). These results collectively indicate that RNASET2 acts as a suppressor to restrict the invasion and metastasis of HCC cells.

Next, we sought to assess the impact of RNASET2 on the proliferation of HCC cells. WB analysis revealed that neither RNASET2 loss nor overexpression influenced the levels of the key cell cycle regulators, cyclin D1 and CDK4 (Figure S2A,B, Supporting Information). Consistent with these findings, flow cytometry assays demonstrated that RNASET2 had only a minimal effect on the progression of the HCC cell cycle (Figure S2C,D, Supporting Information). Additionally, CCK‐8 assays indicated that neither RNASET2 deletion nor overexpression significantly affected the proliferation of HCC cells (Figure S2E,F, Supporting Information).

### 2.2 RNASET2 Deficiency Drives Cholesterol Accumulation in HCC Cells

**Figure 2:**
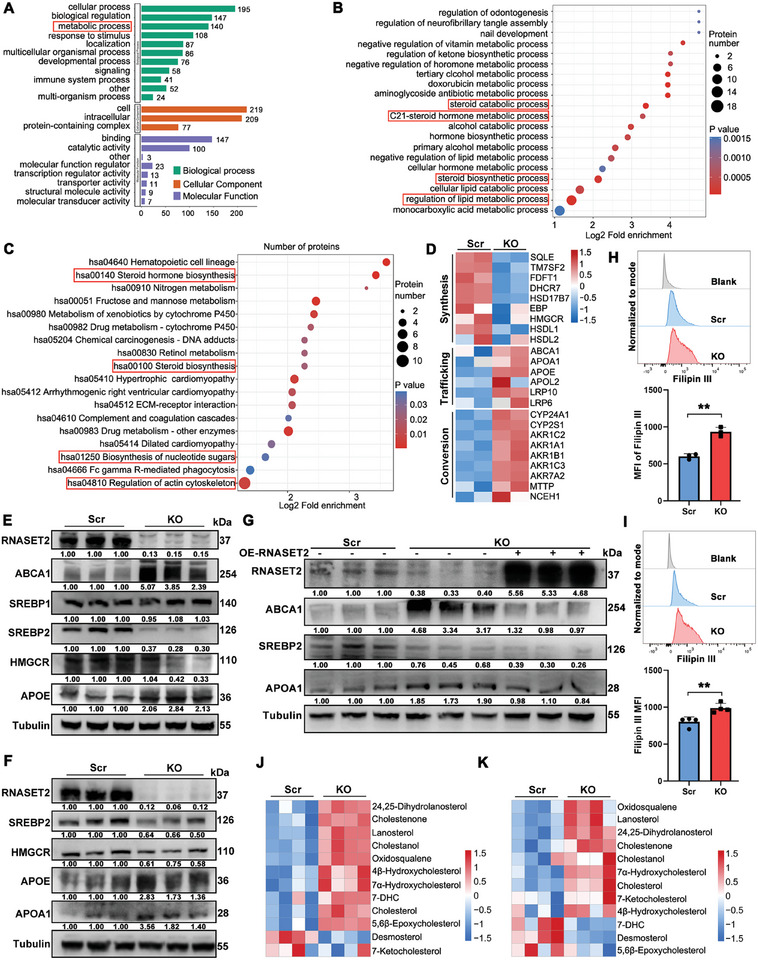
RNASET2 deficiency leads to cholesterol accumulation in HCC cells: A) GO analysis of HuH‐7 cells. Data were defined as log2 (fold enrichment; RNASET2^KO^ versus scrambled control; *n* = 2). B,C) The Biological Process (B) and KEGG (C) analysis of all differentially expressed proteins in proteomic data. Data were defined as log2 (fold enrichment; RNASET2^KO^ versus scrambled; *n* = 2). D) Heatmap of significantly changed proteins associated with cholesterol metabolism in proteomic data. The protein levels were normalized as log2 (fold change; RNASET2^KO^ versus scrambled; *n* = 2). E,F) WB analysis of the levels of ABCA1, SREBP1, SREBP2, HMGCR, APOE, and APOA1 in RNASET2^KO^ and scrambled control HuH‐7 (E) and PLC/PRF/5 (F) cells (*n* = 3). G) WB analysis of the levels of ABCA1, SREBP2, and APOA1 in scrambled, RNASET2^KO^, and RNASET2^KO^/ OE‐RNASET2 HuH‐7 cells. H,I) The levels of free cholesterol in RNASET2^KO^ HuH‐7 (H) (*n* = 3) and PLC/PRF/5 (I) (*n* = 4) cells were stained with Filipin III and tested by flow cytometry. Quantification was shown right. J,K) Relative content of cholesterol and its intermediate metabolites of RNASET2^KO^ HuH‐7 (J) and PLC/PRF/5 (K) cells were detected by UPLC‐MS/MS compared to control cells. The metabolites levels were normalized as log2 (RNASET2^KO^ versus scrambled; *n* = 4). Data are presented as mean ± SD. **p <0.01, by two‐tailed unpaired Student's *t*‐test (H,I).

To investigate the regulatory mechanism of RNASET2 on the invasion and metastasis of HCC cells, we performed an unbiased proteomic analysis using RNASET2^KO^ and control HuH‐7 cells. Our analysis revealed that 100 proteins were significantly upregulated and 137 proteins were downregulated upon RNASET2 deletion (Figure S3A,B, Supporting Information). Furthermore, the Gene Ontology (GO) analysis indicated that RNASET2 deficiency led to differential expression of 140 proteins involved in metabolic processes (**Figure** **2**A), suggesting the possibility of metabolic reprogramming. Notably, the proteomic Biological Process analysis showed a high enrichment of lipid metabolic processes, as well as a significant increase in steroid biosynthetic process, steroid catabolic process, and C21‐steroid hormone metabolic process in the RNASET2^KO^ group (Figure 2B). The KEGG analysis revealed a significant increase in the regulation of actin cytoskeleton pathway after RNASET2 ablation, which is consistent with the observed promotion of HCC metastasis due to RNASET2 deficiency. Interestingly, the KEGG analysis also showed a strong enrichment of the steroid biosynthesis and steroid hormone biosynthesis pathways following RNASET2 deletion (Figure 2C).

Cholesterol is a crucial type of lipids that acts as a precursor for the biosynthesis of bile acids, steroid hormones, and vitamin D3.^[^ ^28^, ^29^, ^30^ ^]^ The proteomic data provided above indicated a potential connection between RNASET2 and cholesterol metabolism. To confirm this association, we analyzed the differentially expressed enzymes involved in cholesterol metabolism. The heatmap analysis revealed that the ablation of RNASET2 inhibited the production of cholesterol synthesis enzymes (such as HMGCR, DHCR7, and HSD17B7), while promoted the production of cholesterol transport enzymes (such as ABCA1, APOE, and APOA1) and cholesterol conversion enzymes (such as AKR1C2, AKR1C3, and AKR7A2) (Figure 2D). Consistently, WB analysis also displayed that the deficiency of RNASET2 impaired the expression of sterol regulatory element‐binding protein 2 (SREBP2), which is a crucial transcription factor for cholesterol biosynthesis, as well as its downstream target HMGCR, the rate‐limiting enzyme of cholesterol biosynthesis (Figure 2E,F; Figure S3C, Supporting Information).^[^ ^29^, ^30^, ^31^, ^32^, ^33^, ^34^ ^]^ Rescue experiments conducted with RNASET2 overexpression on RNASET2^KO^ HCC cells revealed that the RNASET2 ^KO^‐mediated increase in ABCA1 and APOA1 production were impaired (Figure 2G).

As expected, both flow cytometry and cholesterol assay kit analyses demonstrated that the deletion of RNASET2 resulted in elevated cholesterol levels in HCC cells (Figure 2H,I; Figure S3C, Supporting Information). Furthermore, the cholesterol concentration in RNASET2^KO^ HCC cells derived from lung tumor tissues was significantly higher than that observed in the control group (Figure S3D, Supporting Information). Liquid chromatography‐mass spectrometry (LC‐MS/MS) analysis revealed that RNASET2 ablation increased the concentration of cholesterol derivatives, including 4β‐hydroxycholesterol, 7α‐hydroxycholesterol, and 7‐ketocholesterol (Figure 2J,K). This finding was consistent with the elevated expression of cholesterol conversion enzymes in RNASET2^KO^ HCC cells (Figure 2D). Additionally, LC‐MS/MS analysis showed that the loss of RNASET2 resulted in a significant upregulation of cholesterol levels (Figure 2J,K). The accumulation of cholesterol and its metabolites may result in feedback inhibition of the SREBP2/HMGCR‐mediated cholesterol biosynthesis pathway.^[^ ^35^, ^36^ ^]^ This could explain the observed downregulation of cholesterol biosynthesis‐related enzymes in the proteomic and WB results (Figure 2D,E,F; Figure S3E, Supporting Information). Furthermore, we observed an increase in the concentration of several cholesterol precursors, such as oxidosqualene, lanosterol, and 24,25‐dihydrolanosterol, in the absence of RNASET2 (Figure 2J,K). The impaired cholesterol biosynthesis may be responsible for these results. Together, the above data indicate that the ablation of RNASET2 leads to cholesterol accumulation in HCC cells.

Although the regulation of lipid metabolic processes was most enriched in RNASET2^KO^ HCC cells (Figure 2B), the expression levels of proteins involved in the regulation of other lipid metabolism pathways, including fatty acids, triglyceride, phospholipid, and cholesterol uptake, were not significantly altered (Figure S3F, Supporting Information). Consistently, there were no significant changes in the cholesterol uptake enzyme LDLR, as well as the rate‐limiting enzymes for fatty acid biosynthesis (FASN and ACC) in RNASET2^KO^ HCC cells compared to controls (Figure S3G,H, Supporting Information).

Considering the importance of the Warburg effect in the malignancy progression of tumor, we assessed the effect of RNASET2 on glycolysis in HCC. WB analysis revealed slight alteration in the levels of glycolytic enzymes, including pPDK1, Glut4, and LDHA, in both RNASET2 deletion and overexpression HCC cells (Figure S4A,B, Supporting Information). However, our proteomic data confirmed a significant downregulation of the glycolysis rate‐limiting enzyme HK2 in RNASET2^KO^ HCC cells (Figure S4C, Supporting Information). The Seahorse glycolysis stress test demonstrated that RNASET2^KO^ HCC cells exhibited lower glycolysis, glycolytic capacity, and glycolytic reserve compared to controls (Figure S4D, Supporting Information). Furthermore, the invasive and metastatic activities of HCC cells were significantly reduced following treatment with the HK2 inhibitor 2‐DG (Figure S4E,F, Supporting Information), which contrasts with the increased invasive and metastatic activities observed in RNASET2^KO^ HCC cells.

### 2.3 Cholesterol Accumulation Promotes Migration and Invasion of RNASET2‐Deficient HCC Cells

**Figure 3:**
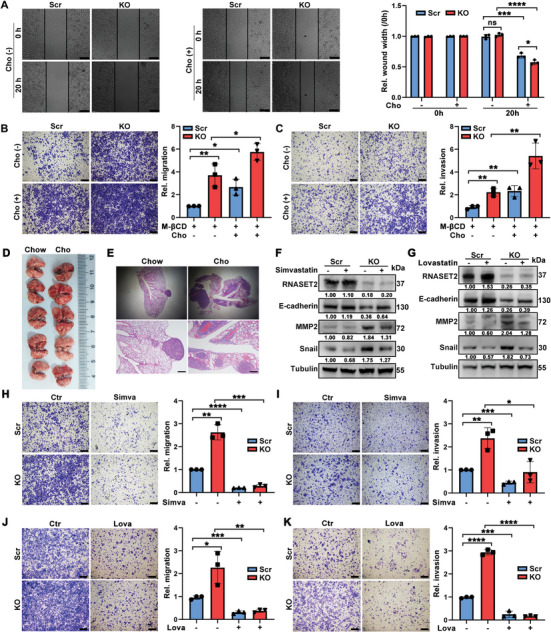
Cholesterol was required for RNASET2^KO^‐mediated migration and invasion of HCC cells: A‐C) Representative images of migration (A,B) and invasion (C) assays of RNASET2 ablation HuH‐7 cells treated in the presence or absence of exogenous cholesterol (*n* = 3). D,E) MHCC97‐H cells intravenously inoculated nude mice were fed with regular chow or 1.25% cholesterol‐supplemented diet for 6 weeks. Macroscopic images of lung metastatic foci (D) (*n* = 5). Representative H&E staining of lung metastasis (E) (*n* = 5). The zoomed images are shown below and the scale bars represented 400 µm. F,G) WB analysis of EMT‐related proteins (MMP2, and Snail) in scr‐ and RNASET2^KO^ HuH‐7 cells treated with 10 µM simvastatin (F) and 3 µM lovastatin (G) for 48 h (*n* = 3). H‐K) Representative images of trans‐well migration (H,J) and invasion (I,K) assays of RNASET2^KO^ HuH‐7 cells supplemented with or without simvastatin (H,I) and lovastatin (J,K) for 48 h (*n* = 3). The distance of the wound gaps was determined using ImageJ. The wound width was normalized against that at time 0 h. Quantification was shown right. Scale bars for wound healing assays: 200 µm, for trans‐well assays: 400 µm. Data are presented as mean ± SD. *p < 0.05, **p <0.01, ***p <0.001,****p <0.0001, *ns* = not significant by two‐tailed unpaired Student's *t*‐test (A‐C and H‐K).

To detect whether cholesterol plays a role in the invasion and metastasis of HCC caused by RNASET2 deficiency, we treated HCC cells with methyl‐β‐cyclodextrin (MβCD) to deplete cholesterol and then supplemented them with exogenous cholesterol to induce cholesterol accumulation (Figure S5A, Supporting Information). As anticipated, the administration of cholesterol facilitated migration and invasion of HCC cells (Figure S5B,C, Supporting Information). Additionally, cholesterol elimination impaired, but cholesterol supplementation significantly accelerated the migration and invasion of both RNASET2^KO^ and scrambled control groups (**Figure** **3**A–C). To assess the impact of cholesterol on HCC metastasis in vivo, nude mice were administered either a standard chow diet or a diet enriched with 1.25% cholesterol immediately following the injection of HCC cells, prior to tumor development. The cholesterol‐enriched diet resulted in a higher number of metastatic tumors in the lungs (Figure 3D,E), suggesting that elevated cholesterol levels may promote metastasis in HCC. Furthermore, incubation with cholesterol biosynthesis inhibitors, simvastatin or lovastatin, significantly inhibited the EMT of HCC cells induced by RNASET2^KO^ (Figure 3F,G). Similarly, trans‐well assays demonstrated that the suppression of entogenous cholesterol concurrently reduced the migration and invasion capabilities of both WT and RNASET2^KO^ HCC cells (Figure 3H–K). Collectively, these findings indicate that the accumulation of cholesterol induced by RNASET2 ablation promotes the migration and invasion of HCC cells.

### 2.4 RNASET2 Orchestrates Glucuronate Metabolism and Cholesterol Elimination in HCC Cells

**Figure 4:**
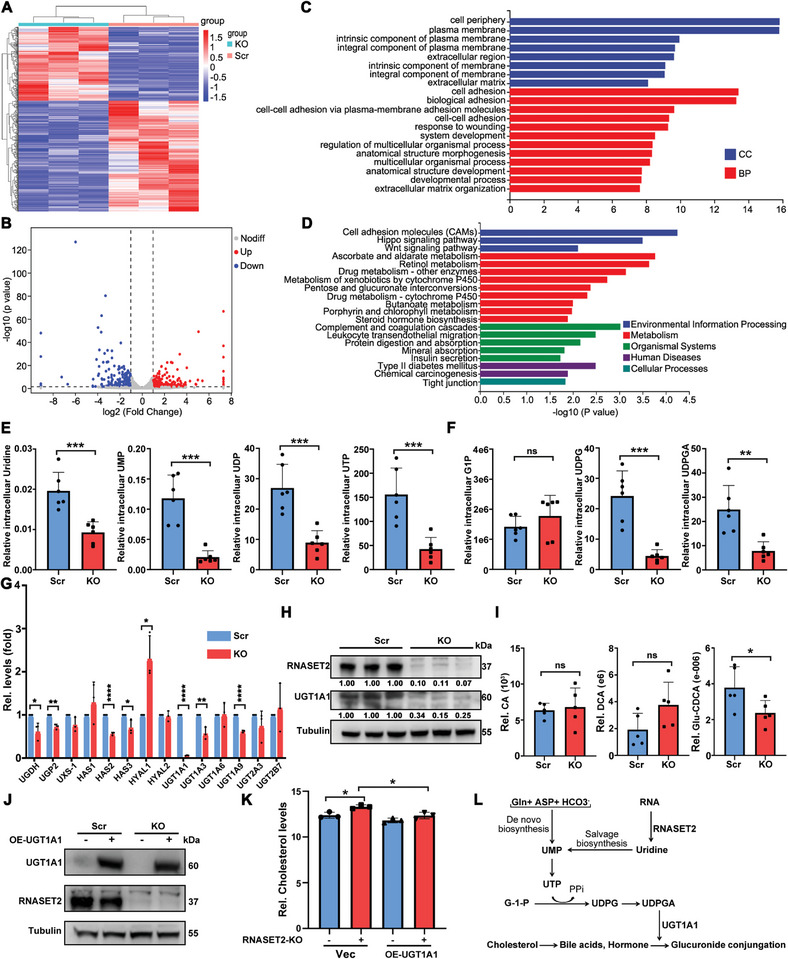
UGT1A1 downregulation is involved in RNASET2 deficiency‐induced cholesterol accumulation. A,B) Heatmap (A) and volcano plot (B) of RNA‐seq data for differentially expressed genes after RNASET2 elimination. Differentially expressed genes were defined as genes with p < 0.01 and log2 (fold change) more (less) than 1 (−1) (scrambled versus RNASET2^KO^; *n* = 3). C,D) GO analysis (C) and KEGG analysis (D) depicting the top enriched pathways based on the significantly altered genes derived from RNA‐seq data (*n* = 3). E,F) LC‐MS assays indicating the levels of pyrimidine salvage biosynthesis related metabolites uridine, UMP, UDP, and UTP (E), and glucuronate metabolism related metabolites G1P, UDPG, and UDPGA (F) of RNASET2^KO^ HuH‐7 cells (*n* = 6). G) qRT‐PCR assessed mRNA levels of enzymes involved in glucuronate metabolism of RNASET2^KO^ HuH‐7 cells (*n* = 3). H) WB analysis of UGT1A1 level in RNASET2^KO^ HuH‐7 cells (*n* = 3). I) LC‐MS measured the levels of bile acids CA, DCA, and glucuronidation‐CDCA (Glu‐CDCA) of RNASET2 deficient HuH‐7 cells (*n* = 5). J) Efficiency of UGT1A1 overexpression (OE‐UGT1A1) in RNASET2^KO^ HuH‐7 cells was identified by WB (*n* = 3). K) Relative cholesterol levels were quantified using Amplex red cholesterol assay kit in RNASET2^KO^ HuH‐7 cells transfected with OE‐UGT1A1 (*n* = 3). L) Schematic depicting the convergence of pyrimidine nucleotide biosynthesis pathway, glucuronic pathway and cholesterol elimination pathway. Gln, glutamine; Asp, aspartate; UDPG, uridine 5′‐diphosphate‐glucose; UDPGA, uridine 5′‐diphosphate glucuronic acid. Data are presented as mean ± SD. *p < 0.05, **p <0.01, ***p <0.001, ****p < 0.0001, *ns* = not significant by two‐tailed unpaired Student's *t*‐test (E‐G, and I) or two‐way ANOVA (K).

To investigate the molecular mechanism underlying cholesterol accumulation caused by RNASET2 ablation, we conducted RNA sequencing (RNA‐seq) on RNASET2^KO^ and control HCC cells. We identified a total of 1412 differentially expressed genes after RNASET2 deletion (**Figure** **4**A,B). GO analysis revealed that RNASET2 deficiency significantly altered membrane and cell adhesion pathway, which supports its role in promoting metastasis (Figure 4C). Additionally, KEGG analysis showed that cell adhesion molecules were the most enriched pathway in RNASET2^KO^ HCC cells, along with enrichment of Hippo and Wnt signaling pathways (Figure 4D). We further examined the expression levels of proteins involved in these pathways and observed only slight alterations, except for β‐catenin (Figure S6A‐C, Supporting Information).

Notably, we also found that RNASET2 deletion significantly enriched multiple metabolism pathways, particularly those related to hepatic biotransformation, such as ascorbate and aldarate, CYP450, pentose and glucuronate, drugs, porphyrin, and steroid hormone biosynthesis, consistent with the proteomic KEGG analysis (Figure 2B,C and Figure 4D). Among the various pathways, we noted the pentose and glucuronate metabolism particularly interesting as it can coordinate the transformation between nucleotide and glucose metabolism. Specifically, ribose‐5‐phosphate (R5P) derived from pentose metabolism serves as a crucial precursor for the biosynthesis of both DNA and RNA.^[^ ^37^, ^38^ ^]^ In addition, glucuronate metabolism converts glucose 1‐phosphate (G‐1‐P) and UTP into UDP‐glucuronic acid (UDPGA), which can conjugate with non‐nutrients, such as bile acids, steroid hormones, and bilirubin, under the catalysis of UDP‐glucuronosyltransferases (UGTs) to facilitate their easy excretion.^[^ ^39^, ^40^, ^41^ ^]^ This process plays a significant role in the excretion of cholesterol in the human body via bile acids.^[^ ^28^, ^40^, ^42^ ^]^ Uridine, a precursor of UTP, has been reported to be released through RNA cleavage by RNASET2.^[^ ^17^, ^18^, ^23^ ^]^ Our LC‐MS/MS assays confirmed that the levels of uridine, UMP, UDP, and UTP were indeed reduced in RNASET2‐deficient cells compared to the control groups (Figure 4E). Additionally, we observed a significant decrease in the intracellular concentrations of UDPG and UDPGA in RNASET2^KO^ HCC cells, while the level of G1P showed a slight increase (Figure 4F).

Next, the expression levels of UGTs in RNASET2^KO^ and WT HCC cells were analyzed. Lower levels of multiple UGTs were observed in RNASET2^KO^ HCC cells, with UGT1A1 being the most downregulated (Figure 4G,H; Figure S6D,E, Supporting Information). UDPGA is a crucial substrate for glucuronidation carried out by UGTs and has previously been confirmed to sustain UGT activity.^[^ ^43^ ^]^ To investigate whether UDPGA influences the expression of UGT1A1, exogenous UDPGA was introduced to RNASET2^KO^ HCC cells, resulting in an increase in UGT1A1 levels (Figure S6F, Supporting Information). LC‐MS/MS analysis showed a notable reduction in the level of intracellular glucuronidated‐CDCA in RNASET^KO^ HCC cells, while the concentrations of un‐conjugated intracellular bile acids CA and DCA were barely altered (Figure 4I). CYP7A1, the rate‐limiting enzyme for converting cholesterol into bile acids,^[^ ^28^, ^29^, ^40^, ^42^ ^]^ showed no significant alteration in transcription and protein levels (Figure S6E,G,H, Supporting Information). Of note, UGT1A1 overexpression restored the cholesterol accumulation caused by RNASET2 deletion (Figure 4J,K). These findings support a model in which RNASET2 deficiency leads to a reduction in uridine, blocking UTP salvage synthesis, thereby attenuating glucuronate metabolism and UGTs expression. Consequently, this results in the failure of cholesterol elimination due to restrained formation of glucuronidated‐bile acid in RNASET2^KO^ HCC cells (Figure 4L).

### 2.5 RNASET2 Restrains the Activation of MET Signaling Pathway in HCC Cells

Cholesterol has been found to play a crucial role in maintaining the stability of RTK family members, such as EGFR and MET, by inhibiting autophagic degradation.^[^ ^12^, ^44^ ^]^ Conversely, the efficacy of several TKIs in HCC is improved by lowering cholesterol through the use of lovastatin, which induce RTK autophagy.^[^ ^44^ ^]^ Additionally, certain members of the RNase A family, including RNase 5, have been identified as ligands to activate RTKs, such as EGFR.^[^ ^20^, ^21^, ^45^ ^]^ The fungus‐derived RNase T2 from *Aspergillus niger* has been reported to compete with RNase 5 and inhibit melanoma metastasis.^[^ ^24^ ^]^ These findings led us to speculate that RNASET2 could suppress HCC cell metastasis by repressing the EGFR signaling pathway through either reducing cholesterol levels or impeding RNase 5‐induced EGFR activation. However, co‐IP analysis revealed no interaction between RNASET2 and EGFR (Figure S7A, Supporting Information). Moreover, the elimination or overexpression of RNASET2 only had a slight effect on EGFR phosphorylation (Figure S7B,C, Supporting Information). Of interest, phosphorylation of the well‐known downstream signaling molecule ERK1/2, which is associated with RTKs, was significantly increased in RNASET2^KO^ HCC cells, and the opposite effect was observed with RNASET2 overexpression (Figure S7B,C, Supporting Information). This suggests that RNASET2 may activate ERK1/2 through another member of the RTKs family.

**Figure 5:**
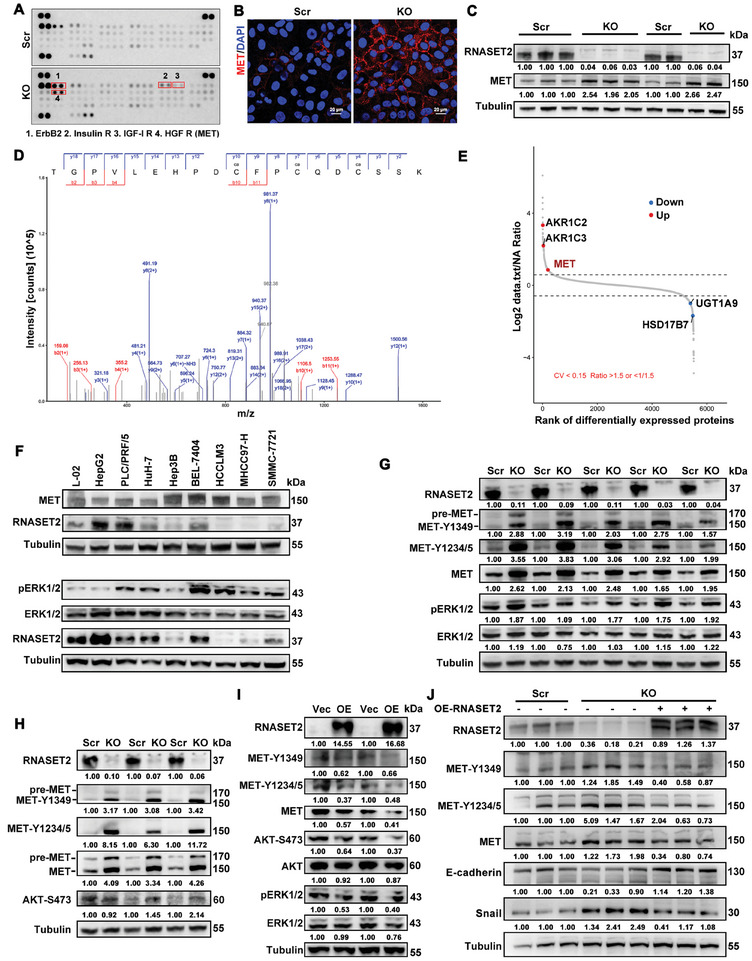
RNASET2 suppresses the activation of MET signaling: A) Human phospho‐RTK antibody array analysis in scr‐ or RNASET2^KO^‐HuH‐7 cells (*n* = 1). B) Immunofluorescent staining of MET in scr‐ or RNASET2^KO^‐HuH‐7 cells. Bars represent 20 µm (*n* = 3). C) WB assays of MET level in scr‐ or RNASET2^KO^‐HuH‐7 cells (*n* = 5). D) MS/MS validation of MET. The fragmentation spectrum of the tryptic MET peptide ‐GPVLEHPDCFPCQDCSSK‐ was shown with annotated y‐ions (blue) and b‐ions (red) (*n* = 2). E) Scatter plot showing the rank of differentially expressed proteins in RNASET2 deletion versus scrambled HuH‐7 cells (*n* = 2). The protein levels were defined as log2 (fold change) more (less) than 1 (‐1). F) WB analysis of the levels of RNAST2, MET, ERK1/2 and pERK1/2 in multiple liver cancer cells (*n* = 3). G‐I) Roles of RNASET2 loss‐ or gain‐of‐function in the levels of the indicated proteins in HuH‐7 (G), PLC/PRF/5 (H) and MHCC97‐H (I) cells were individually identified by WB (*n* = 2 to 5). J) RNASET2 expression was rescued in RNASET2^KO^ HuH‐7 cells, then the levels of the indicated proteins were evaluated (*n* = 3).

We conducted an unbiased antibody array for human phospho‐RTKs with RNASET2^KO^ HuH‐7 cells. The ablation of RNASET2 resulted in an increase in phosphorylation of several members of the RTKs family, including ErbB2, Insulin R, IGF‐I R, and MET. Notably, the phosphorylation of MET (hepatocyte growth factor receptor, HGF R) showed the most significant elevation (**Figure** **5**A). Immunofluorescence, WB, and proteomic assays all confirmed that the deletion of RNASET2 significantly increased the expression of MET in HCC cells compared to the control group (Figure 5B–E; Figure S7D,E, Supporting Information). The levels of MET and phosphorylated ERK1/2 were significantly higher in HCC cell lines with lower RNASET2 expression (Figure 5F). Whereas RNASET2 ablation had no significant effect on the expression and phosphorylation of IGF‐I R, as well as its substrate IRS1 (Figure S7F, Supporting Information). RNASET2 overexpression also had minimal impact on the total and phosphorylated IGF‐I R in MHCC97‐H cells (Figure S7G, Supporting Information). Similarly, RNASET2 deletion had a slight effect on the activation of ErbB2 (Figure S7H, Supporting Information). Therefore, our study primarily focused on understanding the regulatory function of RNASET2 in MET activation.

We observed that RNASET2 ablation led to a significant increase in both total and phosphorylation levels of MET, as well as the phosphorylation of ERK1/2 compared to the control groups (Figure 5G,H). In contrast, RNASET2 overexpression resulted in a reduction in the phosphorylation levels of MET, AKT, and ERK1/2 (Figure 5I). Rescue experiments conducted with RNASET2 overexpression on RNASET2^KO^ HCC cells demonstrated that RNASET2 deletion triggered increase in MET activity and Snail production were obviously abolished, but the decreased production of E‐cadherin was reversed (Figure 5J). Furthermore, we also discovered that β‐catenin, a downstream molecule of MET signaling,^[^ ^46^ ^]^ was upregulated upon MET activation, as well as the phosphorylation of ERK1/2 (Figure S7I, Supporting Information). The increase in MET expression and activation may explain the elevated expression of β‐catenin and the EMT protein MMP9 upon RNASET2 deletion (Figures S6B,C and S7J, Supporting Information). Surprisingly, co‐IP assay showed no interaction between RNASET2 and MET (Figure S7K, Supporting Information). These results suggest that RNASET2 has a contrasting effect on RTKs activation compared to RNase A enzymes, and this effect is not dependent on protein interaction.

### 2.6 Cholesterol Induces both Migration and Invasion in RNASET2‐Deficient HCC Cells through Activating MET

**Figure 6:**
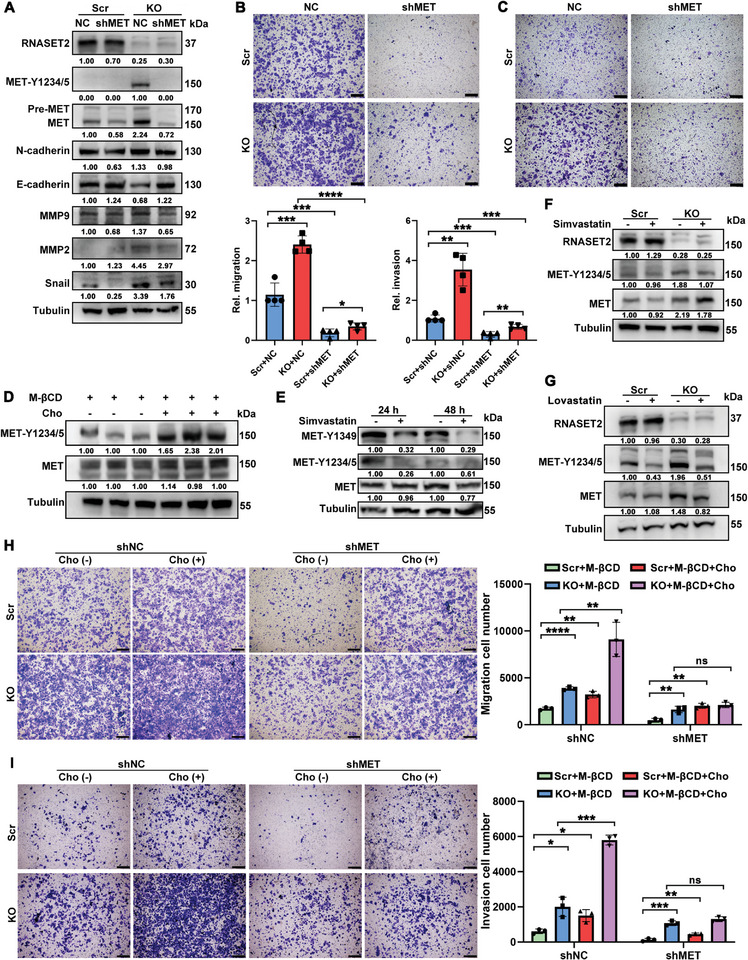
MET is responsible for cholesterol‐induced migration and invasion in RNASET2^KO^ HCC cells: A) WB analysis of the levels of EMT markers including N‐cadherin, E‐cadherin, MMP9, MMP2, and Snail1 in RNASET2^KO^ HuH‐7 cells transfected with shMET (*n* = 3). B,C) Functions of shMET in RNASET2^KO^‐promoted migration and invasion of HuH‐7 cells were confirmed using trans‐well migration assay (B) and invasion assay (C). Quantification was shown below (*n* = 3). D) WB determination of phosphorylated MET in MHCC97‐H cells cultured with or without exogenous cholesterol for 2 h after MβCD treatment (*n* = 3). E) WB detection for MET phosphorylation of MHCC97‐H cells incubated with 10 µM simvastatin for 24 or 48 h (*n* = 3). F,G) WB analysis of MET phosphorylation inhibition by simvastatin (F) or lovastatin (G) in scr‐ and RNASET2^KO^ HuH‐7 cells after 24 h of culture (*n* = 3). H,I) Representative images of migration (H) and invasion (I) assays of RNASET2^KO^ HuH‐7 cells transfected with shMET in the presence or absence of exogenous cholesterol. Quantification was shown right (*n* = 3). Scale bars for trans‐well assays: 400 µm. Data are presented as mean ± SD. *p < 0.05, **p <0.01, ***p <0.001, ****p <0.0001, *ns* = not significant by two‐tailed unpaired Student's *t*‐test (B,C,H,I).

To investigate whether RNASET2^KO^‐mediated promotion in the invasion and metastasis of HCC cells depends on MET activation, we knocked down (KD) *MET* in RNASET2^KO^ HCC cells using shRNAs. We found that the increased expression of mesenchymal markers, including N‐cadherin, ZEB1, MMP9, MMP2, and Snail, triggered by RNASET2 deficiency were abolished following MET inhibition (**Figure** **6**A; Figure S8A, Supporting Information). Moreover, the enhanced migration and invasion capabilities of RNASET2^KO^ HCC cells were hindered by MET interference (Figure 6B,C; Figure S8B, Supporting Information). Savolitinib dramatically repressed the phosphorylation of MET in RNASET2^KO^ HCC cells (Figure S8C, Supporting Information). Accordingly, trans‐well assays demonstrated that savolitinib successfully reversed the promotive effect of RNASET2 deficiency on the migration and invasion of HCC cells by inactivating MET (Figure S8D,E, Supporting Information). These findings suggest that MET signaling is a downstream effect of RNASET2 and plays a critical role in the migration and invasion of HCC driven by RNASET2 deficiency.

Next, we assessed the effect of cholesterol on MET activation. Supplement with cholesterol notably upregulated MET phosphorylation in HCC cells, whereas simvastatin treatment dramatically downregulated MET activation (Figure 6D,E). Additionally, lowering intracellular cholesterol with simvastatin or lovastatin both significantly reversed the RNASET2^KO^‐promoted MET phosphorylation back to a normal state (Figure 6F,G), suggesting an essential role of cholesterol in RNASET2 deletion‐induced MET activation. Subsequently, we investigated whether MET activation was responsible for cholesterol‐triggered migration and invasion in HCC cells. As expected, the promotive role of cholesterol in migration and invasion was largely diminished upon MET silencing (Figure 6H,I; Figure S8F, Supporting Information). Given that cholesterol has been reported to stabilize RTKs by inhibiting autophagic degradation,^[^ ^12^, ^44^ ^]^ we aimed to assess autophagic activity by monitoring the lipidation of LC3B (LC3II) in the context of RNASET2 deficiency. We observed a decrease in LC3II levels in RNASET2^KO^ HCC cells, which was significantly restored following treatment with lovastatin or MβCD to lower intracellular cholesterol levels (Figure S9A‐C, Supporting Information). Concurrently, immunofluorescence assays revealed that RNASET2^KO^ cells exhibited reduced co‐localization (indicated by yellow spots) of LC3B and MET in HuH‐7 cells, a phenomenon that was reversed by cholesterol depletion using MβCD treatment (Figure S9D, Supporting Information). Collectively, these observations indicate that the deletion of RNASET2 can enhance MET activation through the accumulation of cholesterol, thereby inhibiting autophagic degradation.

**Figure 7:**
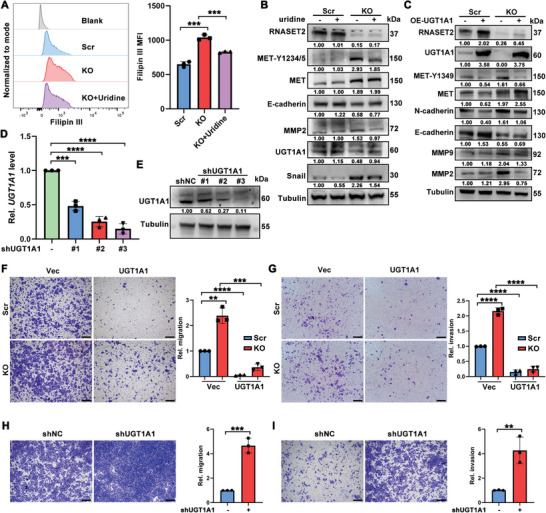
RNASET2 deficiency caused reduction of UGT1A1 promoted HCC metastasis: A) Cholesterol levels of RNASET2^KO^ HuH‐7 cells incubated with uridine (200 µM) for 48 h were measured with filipin III staining. Quantification was shown right (*n* = 3). B) WB assays of the indicated proteins in Scr‐ and RNASET2^KO^ HuH‐7 cells cultured with or without uridine for 48 h. C) The levels of MET phosphorylation and EMT makers production in RNASET2^KO^ HuH‐7 cells transfected with OE‐UGT1A1 (*n* = 3). D,E) The mRNA (D) and protein (E) levels of UGT1A1 in shRNA transfected HuH‐7 cells (*n* = 3). F,G) Representative images of migration (F) and invasion (G) of the indicated subgroups. Quantification was shown right (*n* = 3). Scale bars for trans‐well assays: 400 µm. H,I) Representative images of migration (H) and invasion (I) in the control and shUGT1A1 HuH‐7 cells. Quantification was shown on the right panel (*n* = 3). Scale bars for trans‐well assays: 400 µm. Data are presented as mean ± SD. *p < 0.05, **p <0.01, ***p <0.001, ****p <0.0001 by two‐tailed unpaired Student's *t*‐test (A,D, and F‐I).

Given the significant impact of cholesterol on RNASET2^KO^ induced MET activation and HCC metastasis, we further investigated the role of uridine in cholesterol accumulation resulting from RNASET2 deficiency. The addition of exogenous uridine effectively mitigated the cholesterol upregulation induced by RNASET2^KO^ (**Figure** **7**A). Moreover, uridine administration not only restored the expression of UGT1A1, which was downregulated in RNASET2^KO^, but also inhibited the MET activation and the subsequent production of EMT proteins promoted by RNASET2^KO^ (Figure 7B).

To investigate the potential role of UGT1A1 in modulating MET activation, we overexpressed UGT1A1 in RNASET2^KO^ HCC cells. The results indicated that UGT1A1 overexpression effectively reversed the MET activation induced by RNASET2^KO^ and restored the levels of targeted EMT proteins to baseline levels (Figure 7C). Furthermore, we conducted transfections of HuH‐7 cells with shUGT1A1 (Figure 7D,E). The results demonstrated that UGT1A1 overexpression significantly reduced migration and invasion in both RNASET2^KO^ and control HCC cells, whereas the knockdown of *UGT1A1* enhanced the migratory and invasive capabilities of these cells (Figure 7F–I). Together, these results indicate that MET activation plays a crucial role in the cholesterol accumulation‐promoted migration and invasion of RNASET2^KO^ HCC cells.

### 2.7 Targeting MET Attenuates RNASET2 Deficiency‐Induced HCC Metastasis

**Figure 8:**
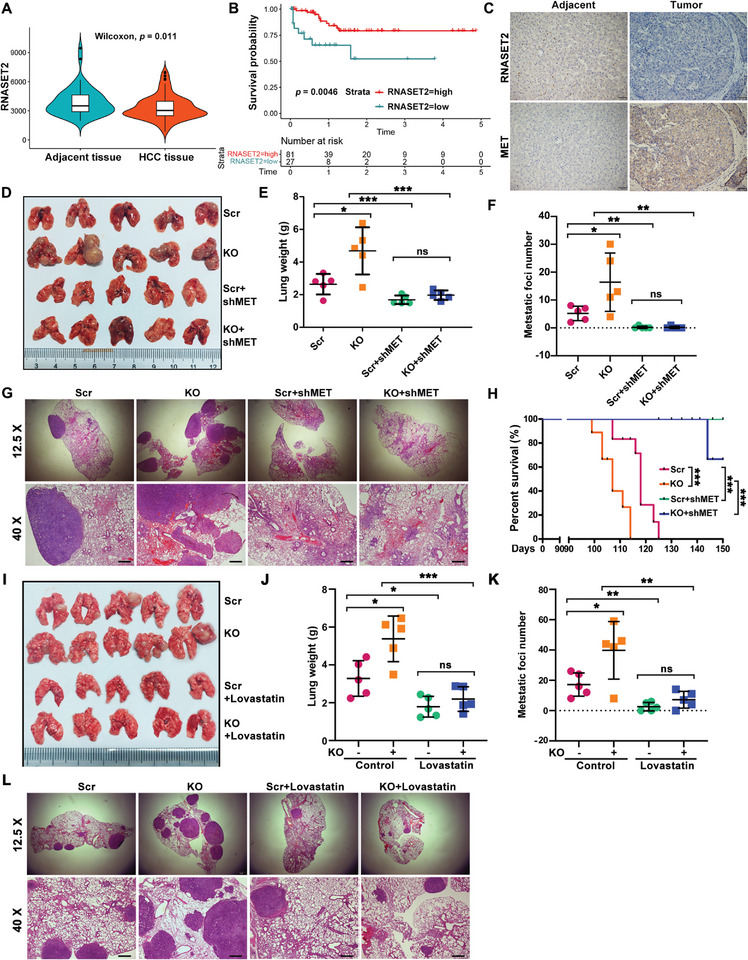
Targeting MET attenuates RNASET2 deletion‐mediated HCC metastasis: A) the level of RNASET2 in tumor tissues versus adjacent tissues of HCC patients from GEO data (GSE76427). B) The survival probability of patients with low and high RNASET2 expression in GSE76427 data. C) Representative IHC staining images of RNASET2 and MET in tumor tissues and matched adjacent noncancerous tissues of HCC patients (*n* = 5). Scale bars, 50 µm. D‐F) Macroscopic views (D), lung weight (E), and metastatic foci number (F) of the mouse models *i.v*. injected with scrambled control, RNAST2^KO^, scrambled‐shMET, and RNAST2^KO^‐shMET HuH‐7 cells (*n* = 5). G) H&E staining of lung metastasis of the indicated groups. The images shown in the lower panel were with higher magnification (*n* = 5). Scale bars: 400 µm. H) Survival of lung metastatic mouse model triggered by the labeled groups (*n* = 8). I–K) Macroscopic views (I), lung weight (J), and metastatic foci number (K) of the lung metastasis mouse model groups: scrambled, RNAST2^KO^, scrambled + Lovastin, and RNAST2^KO^ + Lovastin (*n* = 5). L) H&E staining of lung metastasis of the indicated groups. The images shown in the lower panel were with higher magnification (*n* = 5). Scale bars: 400 µm. Data are presented as mean ± SD. *p < 0.05, **p <0.01, ***p <0.001, ****p <0.0001, *ns* = not significant by two‐tailed unpaired Student's *t*‐test (E,F,J,K), log‐rank (Mantel‐Cox) test (B,H).

To assess the clinical relevance of RNASET2 in patients with HCC, we analyzed RNASET2 levels in both adjacent non‐tumor tissues and tumor tissues of HCC patients using the GSE76427 database. Our analysis revealed a significant decrease in RNASET2 levels within HCC tumor tissues (**Figure** **8**A). Furthermore, patients with low RNASET2 expression exhibited markedly shorter survival probabilities (Figure 8B). Additionally, IHC assays demonstrated that RNASET2 expression was lower in HCC tumor tissues compared to adjacent non‐tumor tissues, but MET expression was found to be elevated in the tumor tissues (Figure 8C). To validate the role of MET in RNASET2 deletion‐triggered HCC metastasis in vivo, we generated lung metastasis models by intravenously injecting MET^KD^/RNASET2^KO^ HCC cells into nude mice. Our findings revealed that RNASET2 deletion alone enhanced lung metastasis of HCC cells, but this effect was significantly reversed upon MET silencing (Figure 8D–F). H&E staining also showed an increased number of lung nodules in mice administered with RNASET2^KO^ HCC cells compared to the control groups. However, MET^KD^/RNASET2^KO^ significantly reduced the number of lung nodules compared to RNASET2 deletion alone (Figure 8G). Mice in the RNASET2^KO^ group also exhibited shorter overall survival than those in the control group, but MET silencing rescued almost all the mice from death (Figure 8H). Considering that RNASET2^KO^ promotes MET activity through cholesterol accumulation, we administered lovastatin to RNASET2^KO^ HCC cells in a mouse model of lung metastasis. We found that targeting cholesterol significantly suppressed RNASET2^KO^‐induced HCC metastasis (Figure 8I–L). Collectively, these data confirm that RNASET2 deficiency induces HCC metastasis via cholesterol‐mediated activation of MET.

## 3 Discussion

RNASET2 is an enzyme that cleaves RNA, resulting in the degradation of transcripts and the production of nucleotide fragments with adenosine/guanosine terminus for G residues and a uridine residue for U residues.^[^ ^17^, ^18^ ^]^ Although previous literature has shown a strong link between RNASET2 and tumor progression,^[^ ^23^, ^25^, ^26^, ^27^ ^]^ its role in the progression of HCC has remained unclear. In this study, we found that the expression of RNASET2 was significantly decreased in HCC and negatively correlated with the metastasis potential of HCC cell lines. The decrease in RNASET2 led to a reduction in the level of the RNA degradation product uridine, which resulted in the inhibition of UTP salvage synthesis and affected glucuronate metabolism. This led to an accumulation of cholesterol and activated the MET‐ERK1/2 signaling pathway, thus promoting HCC metastasis. These findings reveal a novel mechanism of RNASET2 in regulating HCC metastasis, involving UGT‐mediated glucuronate and cholesterol metabolism, RTK signaling, and EMT.

UGTs are crucial enzymes involved in the conjugation of UDPGA with non‐nutrients, such as bile acids, converting them for easy elimination. Among these enzymes, UTG1A1 was found to be most downregulated upon RNASET2 deletion in our current study. The low level of UGT1A1 resulted in a decreased formation of glucuronidated‐CDCA and reduced hepatic biotransformation elimination of cholesterol. Moreover, overexpression of UGT1A1 in RNASET2^KO^ HCC cells partially restored the cholesterol accumulation, thereby partially reversing the promotive effects of RNASET2 deficiency on migration and invasion. Our study provides a novel insight into the coordination between RNA degradation and glucuronic acid metabolism in modulating cholesterol metabolism balance.

UGT1A1 has been extensively studied as a drug‐ or xenobiotic‐metabolizing enzyme, particularly in relation to the elimination of bile acids. Numerous studies have shown that UGT1A1 plays a crucial role in the biotransformation of the chemotherapeutic drug irinotecan, which is used to treat advanced CRC and pancreatic cancer. Therefore, the UGT1A1 genotype is widely used as a guide for prescribing the appropriate dosage of irinotecan.^[^ ^47^, ^48^ ^]^ Additionally, Ou *et al.* found that knocking down RNASET2 significantly increased the sensitivity of CRC cells to 5‐FU by reducing autophagic uracil production.^[^ ^23^ ^]^ Based on these findings, we speculate that deficiency in RNASET2, which leads to downregulation of UGT1A1, may also have a significant impact on the effectiveness of targeted therapies in HCC patients, and further investigation in this area is warranted.

Targeting cholesterol biosynthesis has been widely considered as a potential approach for treating cancer metastasis. However, the effects of statins, inhibitors of HMGCR, on cancer prognosis have been a subject of controversy. On one hand, multiple observational studies have demonstrated that statins have the potential to decrease the risk of cancer and cancer‐related mortality.^[^ ^49^, ^50^, ^51^ ^]^ On the other hand, phase III studies have indicated that statins do not provide any clinical benefits for patients with gastric cancer and HCC.^[^ ^52^, ^53^ ^]^ In our study, both the removal and addition of cholesterol manipulation indicated that exogenous cholesterol induced MET phosphorylation and metastasis of HCC cells, while cholesterol removal suppressed MET activation and blocked RNASET2 deletion‐mediated EMT and invasion. These findings suggest that cholesterol not only directly drives HCC metastasis but also plays a role in activating MET signaling upon RNASET2 ablation. This suggests an alternative targeting pathway to inhibit cholesterol‐induced HCC metastasis, rather than relying solely on statins treatment. Furthermore, the impact of cholesterol on maintaining RTKs stability may explain how RNASET2 deficiency also affects IGF‐I R, Insulin R, and ErbB2 activation.

Human RNases can be categorized into two families: RNase A and RNase T2. Among the RNase A family members, some have been identified as ligands that activate RTKs. For example, RNase 1 has been found to be the ligand for EphA4, which plays a role in maintaining breast cancer stemness.^[^ ^45^ ^]^ Similarly, RNase 5 is a ligand for EGFR, facilitating pancreatic cancer progression and metastasis.^[^ ^20^ ^]^ Additionally, RNase 7 has been confirmed as a ligand for ROS1, enhancing HCC tumorigenesis.^[^ ^21^ ^]^ This raises the possibility that RNASET2 could also act as a ligand for RTKs to promote HCC metastasis. However, our study revealed that RNASET2 has opposing effect on RTKs compared to RNase A enzymes. Furthermore, there was no interaction between RNASET2 and MET displayed in the co‐IP assay. Thus, we identified a suppressive role of RNASET2 in HCC metastasis by blocking the MET signaling pathway, independent on direct protein interaction. The contrasting roles of RNASET2 and RNase A family members in the regulation of RTKs can be attributed to variations in their distribution, genetic conservation, and substrate specificity.^[^ ^19^ ^]^ Notably, RNASET2‐mediated RNA cleavage predominantly occurs in lysosomal compartments,^[^ ^17^, ^23^ ^]^ whereas RNase A family enzymes primarily function as secretory ligands that activate RTKs.^[^ ^20^, ^21^, ^45^ ^]^ Further investigation is needed to determine the roles of low levels of serum RNASET2 in HCC patients.

It is well recognized that uridine and other nucleotide metabolites play a significant role in promoting cancer progression by enhancing DNA replication.^[^ ^54^, ^55^ ^]^ Recent studies have also shown that uridine serves as a fuel for both cancer and immune cells in the conditions where nutrients are limited.^[^ ^56^, ^57^ ^]^ However, Diehl *et al.* reported that an excess of individual nucleotides can hinder cell proliferation by disturbing the balance of nucleotide bases required for DNA replication.^[^ ^58^ ^]^ Furthermore, studies have shown that uridine can inhibit the proliferation, invasion, and migration of HCC cells by activating the ferroptosis pathway.^[^ ^59^ ^]^ Our research demonstrates that the loss of RNASET2 reduces the level of uridine, a degradation product of RNA, which hinders the elimination of glucuronidated bile acids through biotransformation. This ultimately leads to cholesterol accumulation and HCC metastasis. The contradictory effects of uridine may be attributed to various factors such as the microenvironment and uridine levels. Therefore, it is important to determine the precise regulation of the diverse roles of uridine.

In conclusion, our current research has revealed that a deficiency in RNASET2, which is responsible for RNA degradation, leads to a reduction in its metabolite uridine. This reduction in uridine subsequently decreases the levels of UTP by inhibiting salvage synthesis. As a result, the metabolism of glucuronate and the expression of UGT1A1 are also inhibited, leading to a failure in excreting cholesterol through UDPGA‐conjugated bile acids. Consequently, cellular cholesterol accumulation activates the MET‐ERK1/2 signaling pathway, promoting the invasion and metastasis of HCC cells. This finding suggests a novel regulation mechanism for MET signaling, involving the convergent regulation of nucleic acids, glucuronic acids, and cholesterol metabolic reprogramming. Our study opens up avenues for further investigation into the interplay between RNA homeostasis and other metabolic reprogramming processes, as well as a potential therapeutic strategy for targeting RTKs in HCC treatment.

## 4 Experimental Section

### Human Samples

Paraffin‐embedded and frozen tumor tissues, along with matched adjacent tissues were retrospectively obtained from male HCC patients who underwent curative resection at Xinqiao Hospital. Plasma specimens (100 µL) were also collected retrospectively from both HCC patients (*n* = 30) and healthy volunteers (*n* = 7). The information of all participants is summarized in Table S1 (Supporting Information). Considering that the incidence of HCC is significantly higher in men than in women,^[^ ^1^, ^2^ ^]^ only male participants were included in this study. All human experiments were approved by the Ethics Committee of Xinqiao Hospital, Army Medical University (ethical number: 2024‐YD 100).

### Cell Lines and Treatment

Human L‐02, BEL‐7404, and SMMC‐7721 cell lines were purchased from the Shanghai cell bank. PLC/PRF/5, HepG2, HuH‐7, Hep3B, MHCC97‐H, and HCCLM3 were obtained from the cell bank of National Collection of Authenticated Cell Cultures. All cell lines were tested for Mycoplasma and preserved at early passages. They were cultured in complete DMEM medium (C11995500BT, Gibco) supplemented with 10% FBS (A3161002C, Gibco) and maintained in a humidified incubator with 5% CO_2_ at 37°C. For TGF‐β treatment, serum‐starved HCC cells were incubated with TGF‐β1 (HZ‐1011, Proteintech) at concentrations of 0, 10, 25, and 50 ng mL^−1^ for 48 h. For HGF (HY‐P70627, Med Chem Express) administration, serum‐starved HCC cells were incubated with HGF at concentrations of 0, 10, and 25 ng mL^−1^ for 15 min. For exogenous cholesterol stimulation, HCC cells rinsed with serum‐free medium were pre‐treated with 0.75% MβCD (C4555, Sigma‐Aldrich) for 2 h. Then, the cells were transferred to fresh serum‐free medium with 0.1% MβCD in the absence or presence of 3 µg mL^−1^ cholesterol (C3045, Sigma‐Aldrich) for another 2 h. Serum‐starved HCC cells were treated with simvastatin (S6196, Sigma‐Aldrich) at a concentration of 10 µM for the indicated time.

### Animal Studies

Male BALB/c nude mice, aged five weeks, were purchased from Vitalriver Laboratory (Beijing, China) and bred in laminar flow cabinets under specific pathogen‐free conditions. Animals were randomly assigned into each experimental group. For the lung metastasis model, 1 × 10^6^ MHCC97‐H cells (vector or OE‐RNASET2) or 2 × 10^6^ HuH‐7 cells (scrambled, RNASET2^KO^, scrambled/shNC, scrambled/shMET, RNASET2^KO^/shNC, RNASET2^KO^/shMET) were intravenously inoculated into the tail vein of each nude mouse (*n* = 5) for the indicated duration. After 7 days, the tumor‐bearing mice were randomly divided into two groups (*n* = 5) and administered either lovastatin (30 mg kg^−1^ in PBS) or PBS by oral gavage every two days until the mice were sacrificed. The lung metastasis was confirmed by hematoxylin‐eosin (H&E) staining. For the peritoneal metastasis mouse model, 1 × 10^6^ HuH‐7 cells (scrambled or RNASET2^KO^) were intraperitoneally inoculated in each nude mouse (*n* = 5). Tumors were extracted at the indicated time points. In experiments involving a cholesterol‐supplemented diet, mice bearing the MHCC97‐H cell inoculated lung metastasis model were divided into two groups: one group was fed a regular chow diet, while the other received a diet supplemented with 1.25% cholesterol for a duration of six weeks. All animal experiments were approved by the Ethics Committee of Xinqiao Hospital, Army Medical University (ethical number: AMUWEC20245241).

### Western Blot

Cell and tissue samples were lysed using RIPA buffer (P0013B, Beyotime) supplemented with protease and phosphatase inhibitors. Total lysates were collected and the protein concentration was quantified using the BCA assay kit (23228, Thermo Fisher). Approximately 20 µg of total protein per lane was then subjected to WB analysis with the specified antibodies. The chemiluminescence signals were detected using enhanced chemiluminescence (38555, Thermo Fisher). At least three independent experiments were conducted and representative results are presented. Actin, GAPDH or Tubulin was utilized as an internal control. Immunoblots were visualized on ImageQuant LAS 4010 (GE Healthcare).

### Histology and Immunohistochemistry

For histological evaluation of mouse lung metastasis, lung tissues of experimental mice were fixed in 4% paraformaldehyde for 24 h. Next, the tissues were embedded in paraffin. Paraffin‐embedded lung tissues were prepared as 4 µm slices and stained with H&E. For immunohistochemistry, paraffin‐embedded tumor and matched adjacent tissues from patients with HCC were prepared as 4 µm slices. The sections underwent antigen retrieval, endogenous peroxidase elimination, and goat serum blocking in sequential order. Afterwards, the slices were stained with RNASET2 antibody (13753‐1‐AP, Proteintech). Brightfield images were acquired using an ortho microscope (Olympus BX63).

### ELISA for RNASET2

Whole blood was collected from HCC patients (*n* = 30) and healthy volunteers (*n* = 7). Plasma was obtained by centrifuging at 1200g for 5 min at 4°C. The concentration of RNASET2 in human plasma samples was determined using the Human RNASET2 ELISA Kit (NBP3‐06926, Novus). The standard and samples were diluted as per the manufacturer's instructions and added to pre‐coated 96‐well plates. After incubating for 1 h at 37°C, the plates were washed and incubated with streptavidin‐conjugated HRP for another hour at 37°C. Following this, peroxidase substrate was added and incubated for 30 min at room temperature. The reaction was stopped with a stop solution, and the optical density was measured at 450 nm. All data were normalized to the RNASET2 concentration.

### Real Time Quantitative PCR (RT‐qPCR)

Total RNA was extracted from HCC cells with the indicated treatment using RNAiso Plus (9109, Takara). The purity and concentration of the RNA were assessed using a NanoDrop One (Thermo Fisher Scientific). The RNA was then reversely transcribed to cDNA using the PrimeScript Real‐Time Master Mix (RR047A, Takara). The mRNA levels were determined by performing qPCR on the CFX96 system (Bio‐Rad) with SYBR Green PCR Master Mix (B21202, Bimake), using 1 µg of cDNA as a template. The relative quantification of target gene expression was performed using the 2^−ΔCt^ method, with *Actin* serving as the internal control. The primers are listed in Table S2 (Supporting Information).

### Co‐Immunoprecipitation (co‐IP)

The washed protein A/G magnetic beads (P2055, Beyotime) were incubated with primary antibodies (EGFR, MET, Flag) or normal rabbit IgG at 4°C for 30 min. Cells were harvested using IP lysis buffer (P0013, Beyotime) with a protease inhibitor cocktail. Total cell lysates were then added to the mixture of antibodies and magnetic beads that had been washed, and the incubation continued at 4°C overnight. The proteins that interacted with the magnetic beads were captured, washed, and treated with SDS loading buffer before being assessed by WB.

### Lentiviral Transfection

For the CRISPR‐Cas9‐based editing of human RNASET2 knockout, sgRNAs from a previous study^18^ were used and assembled into lentiviral constructs with the pU6‐EF1a‐Cas9‐FLAG‐P2A‐EGFP vector. HepG2, HuH‐7, and PLC/PRF/5 HCC cells, which exhibit high expression of RNASET2, were seeded at a density of 5 × 10^4^ cells per well in a 12‐well plate one day prior to transfection. The cells were transfected with sgRNASET2 and scrambled lentiviral constructs following the manufacturer's instructions. After 24 h, fresh culture medium was added to the cells, which were then incubated for an additional 48 h. Subsequently, the cells were screened with puromycin for 2 weeks to select successfully transfected cells. GFP‐positive single cells were sorted into 96‐well plates using the Beckman Coulter MOFLO XDP. Each monoclonal cell was expanded in a 5% CO_2_ incubator at 37°C. The effect of RNASET2 deletion in monoclonal cells was identified by WB.

RNASET2 overexpression was achieved by cloning the full‐length CDS gene (GeneID: 8635; GenBank: NM_003730) into the pFLAG‐CMVi‐puromycin vector. MET knockdown was achieved by subcloning *MET* shRNA (targeted sequences: CAGAATGTCATTCTACATGAG) into the pGCSIL‐hU6‐CMV‐Neomycin vector. UGT1A1 overexpression was achieved by constructing the full‐length CDS gene (GeneID: 54658; GenBank: NM_000463) into the pGC‐FU‐Ubi‐SV40‐blasticidin vector. To establish *UGT1A1* knockdown, UGT1A1 shRNA was introduced, utilizing targeted sequences: #1, CTGGCTGTTTAGAAGTGACTT; #2, CCCACTGTATTCTTCTTGCAT; and #3, CGGGTGAAGAACATGCTCATT, incorporated into the hU6‐MCS‐CBh‐gcGFP‐IRES‐puromycin vector. The validation of all plasmids was performed through DNA sequencing, and they were then assembled into lentiviral constructs. Lentiviral transfection and screening of cell lines were conducted following the aforementioned protocol.

### Human Phospho‐RTK Antibody Array

The Human Phospho‐RTK Array Kit (ARY001B, R&D Systems) was utilized to assess the potential activation of RTK signals in the presence of RNASET2 deficiency. All procedures were performed in accordance with the manufacturer's instructions. Initially, scrambled and RNASET2^KO^ HuH‐7 cells were washed with cold PBS and then solubilized in lysis buffer containing protease inhibitors, while being kept on ice for a duration of 30 min. The protein concentrations were quantified using a BCA assay kit. Array membranes were placed in blocking buffer for 1 h at room temperature. Subsequently, cell lysates (300 µg) were added and allowed to incubate with the array membranes overnight at 4°C. Following three washes, the array membranes were incubated with anti‐phospho‐tyrosine‐HRP detection antibody at room temperature for 2 h. Finally, the activation of RTK signals was measured using chemi reagent mix and ImageQuant LAS 4010 (GE Healthcare).

### Confocal Microscopy

Cells grown on coverslips were fixed in 4% paraformaldehyde for 15 min at room temperature, and permeabilized with 0.1% Triton X‐100 for 15 min. Subsequently, they were blocked with 1% BSA for 1 h at room temperature. Following this, the cells were incubated overnight at 4°C with c‐Met antibody (25869‐1‐AP, Proteintech), LC3B antibody (83506, Cell Signaling Technology). Afterwards, the cells were incubated with the secondary antibody, Alexa Fluor 555‐labeled Goat Anti‐Rabbit IgG (H+L), and Alexa Fluor 488‐labeled Goat Anti‐mouse IgG (H+L) for 1 h at room temperature. Nuclei were stained with DAPI, which was included in the mounting reagent. Immunofluorescence images were captured using a Zeiss LSM 880 equipped with Airyscan, GaAsP PMT detectors, and 405/488/543/647‐nm lasers running Zen v.3.4.

### Cell Migration Assay

For wound‐healing migration assays, the scratch inserts were adsorbed onto 24‐well plates. Subsequently, the HCC cells were evenly seeded into both sides of the inserts using high‐glucose DMEM medium containing 10% FBS. The cell incubator was set at 5% CO_2_ and maintained at 37°C. After 24 h, the inserts were removed and replaced with fresh serum‐free DMEM medium. The migration distance (gap area) was then measured at the indicated times using an inverted microscope (Olympus IX83) at 100 × magnification.

For trans‐well chamber migration assays, 5 × 10^4^ HCC cells with indicated treatments in serum‐free culture medium were inoculated into the upper chamber. The lower chamber contained 700 µL medium with 20% FBS. After incubation for the indicated times, the trans‐well chambers were removed. Non‐invaded cells on the inside membranes were gently removed using cotton swabs, while the penetrated cells on the underside membranes were fixed with 4% paraformaldehyde and stained with 0.1% crystal violet. Images were captured using an inverted microscope (Olympus IX83) at 40 × magnification.

### Cell Invasion Assay

The 8‐µm pore size filter trans‐wells (353097, Corning), coated with a diluted matrigel matrix gel (356234, Corning), were inserted into 24‐well plates. HCC cells (5 × 10^4^) in serum‐free culture medium, along with the indicated treatments, were added to the upper chamber. The lower chamber was filled with culture medium containing 20% FBS. The cell incubator was set at 5% CO_2_ and maintained at 37°C. After incubation for the indicated times, the invaded cells were fixed and stained with crystal violet. Images were captured using an inverted microscope (Olympus IX83) at 40 × magnification.

### Proteomic Analysis

RNASET2^KO^ clones and scrambled HuH‐7 cells were cultured individually in 10 cm dishes with 2 replicates. The cells were washed twice with pre‐cooled PBS when they reached ≈80% confluence. Subsequently, the cells were rapidly collected using pre‐cooled PBS on ice. After centrifugation, the cell precipitates were quickly frozen using liquid nitrogen and transported to Jingjie Biological Company on carbon dioxide ice for proteomic analysis. The cell pellets were then subjected to lysis buffer containing 8 M urea, 1% protease inhibitor, and 1% phosphatase inhibitor. The mixture was sonicated and centrifuged, and the resulting supernatant was transferred to a new Eppendorf tube. The protein concentration of each sample was determined using a BCA assay kit. TCA was slowly added to each protein sample to achieve a final concentration of 20%. The mixture was then incubated at 4°C for 2 h. After centrifugation, the pellets were washed twice with pre‐cooled acetone and subsequently redissolved with 200 mM TEAB in an ultrasonic bath. The protein samples were then digested with trypsin at a ratio of 1:50 (trypsin: protein, m/m) overnight at 37°C.

The digested peptides were loaded with mobile phase A, which contained 0.1% formic acid and 2% acetonitrile. Separation was performed using the EASY‐nLC 1200 ultra‐high performance liquid system. Mobile phase B consisted of 0.1% formic acid and 90% acetonitrile. The liquid phase gradient was as follows: 0 min, 8%–23% B; 68 min, 23%‐32% B; 82 min, 32%‐80% B; 86 min, 80% B. The flow rate was maintained at 500 nL min^−1^. Subsequently, the peptides were loaded into an NSI ion source for ionization and analyzed using Orbitrap Exploris™ 480 mass spectrometry. The ion source voltage was set at 2.3 kV, and the FAIMS compensation voltage (CV) was ‐45 V and ‐65 V. The parent peptide ions and their secondary fragments were detected and analyzed using high‐resolution Orbitrap. The primary MS scanning range was set to 400 m/z with a scanning resolution of 60 000. The secondary MS scanning range was fixed at 110 m/z with a scanning resolution of 15 000. The TurboTMT was turned off. Data acquisition was performed using a data‐dependent scanning (DDA) procedure, where the 25 peptide parent ions with the highest signal intensity were selected for sequential entry into the HCD collision pool after the primary scanning. A fragmentation energy of 27% was used for fragmentation. To improve the effective utilization of the mass spectrum, the automatic gain control (AGC) was set to 100%, the signal threshold was set to 5E4 ions/s, the maximum injection time was set to Auto, and a dynamic exclusion time of 20 s was implemented for series mass spectrum scanning to avoid repeated scanning of parent ions. The resulting data were processed using MaxQuant search engine (v.1.6.15.0).

### Cholesterol Detection with Assay Kit

Cellular cholesterol content was quantified using the Amplex Red cholesterol assay kit (A12216, Thermo Fisher) according to the manufacturer's instructions. Initially, cells were lysed with IP lysis buffer and the resulting supernatants were incubated with a reaction mix. This mix contained horseradish peroxidase (HRP), cholesterol oxidase, and cholesterol esterase, and was incubated for 30 min at 37°C. Fluorescence was then measured using a fluorescence microplate reader with excitation at 560 nm and emission detection at 590 nm.

### Cholesterol Detection by Flow Cytometry

HCC cells were fixed using 2% paraformaldehyde for 5 min, followed by neutralization with 1.5 mg mL^−1^ glycine solution for 10 min. Subsequently, the cells were stained with filipin III at a concentration of 50 µg mL^−1^ for 45 min at room temperature, thereafter they were analyzed using flow cytometry (BD Fortessa).

### Determination of Sterol by LC‐MS

RNASET2 ablation and scrambled HCC cells were individually seeded in 10 mm^2^ dishes (*n* = 6) for 48 h. After washing twice with precooled PBS, the cells were rapidly harvested on ice. Deuterated sterol internal standards were added, and the mixed samples were extracted with 3 mL of DCM: methanol (1:1, v/v) containing BHT at 50 µg mL^−1^. Next, the extracted supernatant from each sample was hydrolyed with 300 µL of 10 M KOH. Each sample was added with 3 mL of DPBS to isolated the organic (lower) layer. Samples were dried under N_2_ and dissolved in 1 mL of hexane. Sterols were isolated using 3 mL aminopropyl SPE columns (Agela Technologies). Finally, the samples were dried using N_2_ and reconstituted with 100 µL of 90% v/v methanol in water.

Samples were resolved by Waters UPLC Acquity‐AB SCIEX QTRAP 6500 using a binary solvent system and gradient elution is performed on a Waters BEH‐C18 (1.7 µm × 100 mm) column with a flow rate of 0.25 mL min^−1^ at 30°C. The mobile phase A was Methanol with 5 mM ammonium acetate, and B was 15% High Purity water in methanol with 5 mM ammonium acetate. The liquid phase gradient condition was optimized as follows: 0 min at 52% B, 5 min from 52 to 78%, 20 min at 78%, 23 min from 78 to 100%, 29 min at 100%, and maintained at 52% mobile phase B for additional 2 min for re‐equilibration.

The MS is operated in multiple reaction monitoring (MRM) mode in positive ESI source. The MS condition was as follows: CUR: 20.00, IS: 5500.00, TEM: 600.00, GS1: 50.00, GS2: 50.00, CAD: 8.00, EP:10.00, DP and CE are variable depending on MRM pair. ESI with transitions optimized for each sterol of interest. Sterols are quantified using the internal standards, surrogate, and relative response factor (RRF) of each sterol of interest. All data were acquired utilizing Analyst 1.6.3 software.

### Determination of Nucleotides, Bile Acids, and UDPGA

Metabolite analysis was conducted as per previous research (PMID: 8949913, PMID: 22498707). Briefly, to extract metabolites from samples, 400 µL of cold methanol/acetonitrile (1:1, v/v) extraction solvent was added to remove the protein and extract the metabolites, then adequately vortexed. For the quantification of the metabolites, solutions of Uridine‐^15^N_2_ (793809, Sigma‐Aldrich), d4‐cholic acid (330256P, Sigma‐Aldrich) were added as internal standards (IS) before sample extraction. The mixture was collected into a new centrifuge tube, and centrifuged at 14 000 g for 20 min at 4°C to collect the supernatant. The supernatant was dried in a vacuum centrifuge. Analyses using multiple reaction mode (MRM) were then performed using an Exion UHPLC coupled to a QTRAP 6500 plus (AB Sciex). The analytes were separated on a Waters BEH Amide column (2.1 mm × 100 mm, 1.7 µm) and the temperature was set at 35°C. The injection volume was 2 µL and samples were kept at 4°C through the analysis. Mobile phase A: 90% H_2_O + 2 mM ammonium formate + 10% acetonitrile, mobile phase B: 0.4% formic acid in methanol. An optimized gradient (85% B at 0 min, 80% B at 3 min, 70% B at 6 min, 50% B at 10.5 min, 85% B at 15.6 ‐23 min) was established with a flow rate of 0.3 mL min^−1^. Peak intensity of target analytes was normalized to IS for comparison.

### RNA‐Sequencing

RNASET2 deficiency and scrambled HepG2 cells were cultured individually in T75 cell culture bottles with 3 replicates. Cells were harvested using RNAiso Plus reagent when they reached 70% confluence. Total RNA samples were extracted and quantified by Agilent 2100 Bioanalyzer. The mRNA with Poly(A) structure was isolated from total RNA using Oligo(dT) Magnetic beads to construct dual‐indexed cDNA libraries. Next, cDNA libraries ≈400 bp were enriched by PCR amplification and quantified by Agilent 2100 Bioanalyzer. Subsequently, RNA‐seq was performed using an Illumina Novaseq 6000 platform at Personal Biotechnology Co., Ltd (Shanghai, China). HTSeq statistics were used to compare the read count value of each gene as the raw expression data. Fragments per kilobase of transcript per million (FPKM) were used to normalize gene expression. Additionally, differential expression analysis was conducted using DESeq2. Genes with a significant *p* < 0.05 and log_2_ fold change > 1 were considered as differentially expressed genes.

### Statistics

All reported sample sizes (n) represented biological replicates or independent experiments. Quantification methods and statistical analyses for the proteomics, RNA‐sequencing and LC‐MS/MS data were described in the respective sections of the STAR Methods. Additional statistical analysis was performed using GraphPad Prism 8.0.2 software. The experimental data were presented as mean ± SD. For two groups, a two‐tailed unpaired Student's *t*‐test was used to compare the data. For multiple groups and tumor growth studies, analysis was conducted using two‐way ANOVA. For Kaplan‐Meier survival curves, the log‐rank (Mantel‐Cox) test was performed.

### Ethics Approval and Consent to Participate

All samples were obtained after obtaining written informed consent. The use of these clinical specimens in the study was approved by the Ethics Committee of the Xinqiao Hospital of Army Medical University (ethical number: 2024‐YD 100). Animal studies were conducted following the guidelines approved by the Institutional Animal Care and Use Committee of Army Medical University (ethical number: AMUWEC20245241).

## Conflict of Interest

The authors declare no conflict of interest.

## Author Contributions

Y.X. and Y.C. contributed equally to this work. Y.L. and Y.X. designed the project. Y.X. performed experiments with the help of Y.C., J.Z., J.W., and S.Y. . LC‐MS/MS assays of metabolites were performed by Y.X. and J.Z. . Y.X., Y.C. H.Z., and L.W. analyzed the data. J.L., Y.Z., J.P., L.J., Q.C., and R.X. provided technical support. Y.L., Y.X., Y.C., and J.Z. interpreted the data. Y.L., Y.X., and Y.C. wrote the manuscript; Y.L. supervised this project. All authors reviewed the manuscript and provided comments and suggestions.

## Supporting information



Supporting Information

## Data Availability

The data that support the findings of this study are available from the corresponding author upon reasonable request.
