# Supplementary material for: RNASET2 Deficiency Induces Hepatocellular Carcinoma Metastasis through Cholesterol‐Triggered MET Activation
Source: Adv Sci (Weinh). 2025 Feb 4;12(12):2411888. doi: 10.1002/advs.202411888 (PMC11948071; doi:10.1002/advs.202411888)
Supplement: Supplementary file 1 — Supporting Information [file ADVS-12-2411888-s001.docx]

**Supporting Information**

**RNASET2 deficiency induces hepatocellular carcinoma metastasis through cholesterol-triggered MET activation**

*Yanquan Xu, Yu Chen, Jiangang Zhang, Jingchun Wang, Shuai Yang, Huakan Zhao, Lei Wu, Juan Lei, Yu Zhou, Jin Peng, Lu Jiang, Qian Chen, Rong Xin, Jianyun Zhou, and Yongsheng Li*^*^

**
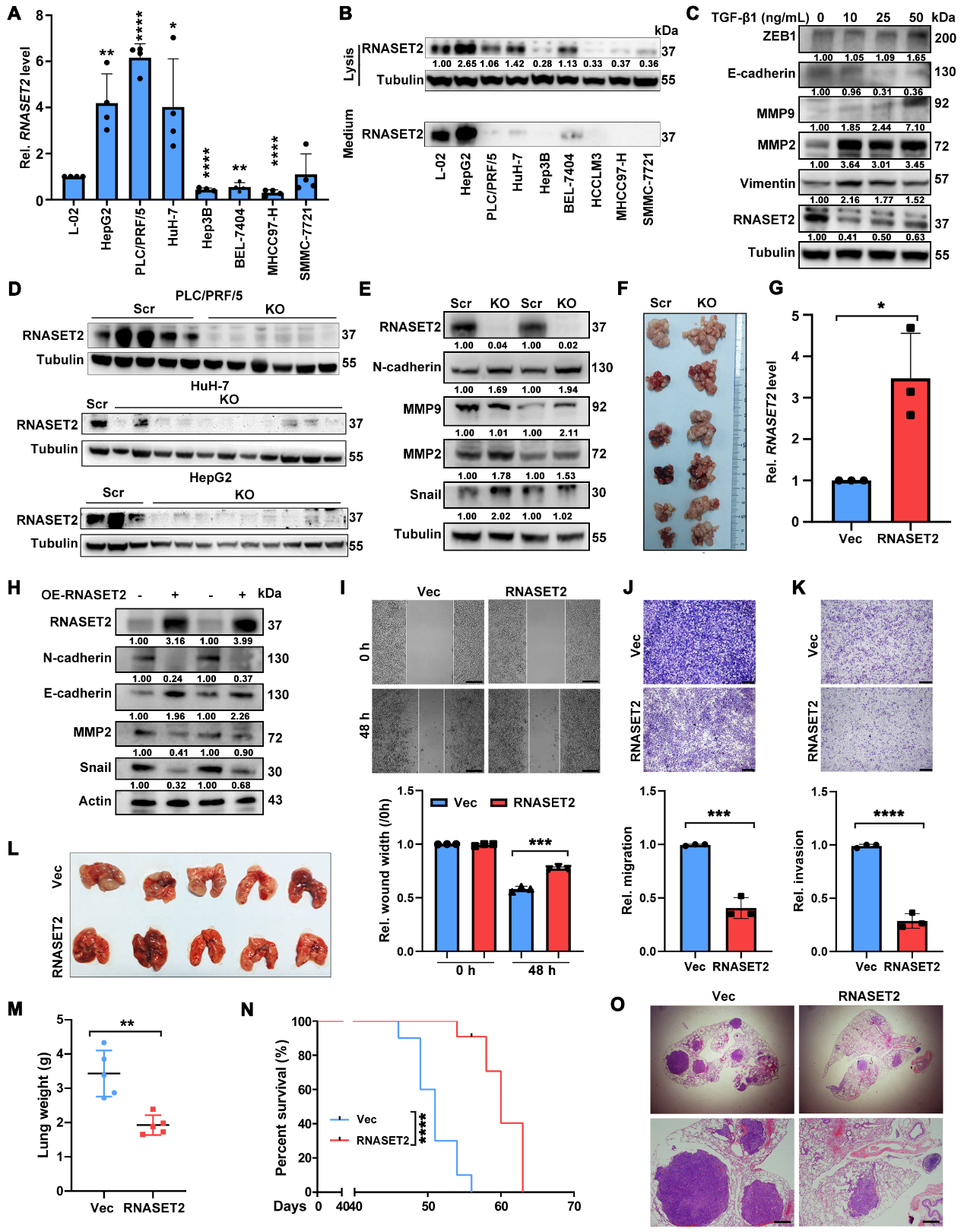
**

**Figure S1.** RNASET2 attenuates HCC invasion and metastasis: A,B) RNASET2 mRNA (A) and protein (B) levels in multiple liver cancer cell lines were evaluated by qRT-PCR and WB respectively (n = 4). C) HCCLM3 cells were incubated with the indicated concentrations of TGF-β1 for 48 h. The levels of RNASET2 and EMT-related proteins (ZEB-1, E-cadherin, MMP9, MMP2, and Vimentin) were assessed with WB (n = 3). D) Stable RNASET2 knockout PLC/PRF/5, HuH-7 and HepG2 monoclonal cell lines constructed by CRISPR-Cas9 lentiviral (sgRNASET2, RNASET2^KO^) and scrambled controls (Scr) were confirmed via WB. E) Quantification of EMT-related proteins in RNASET2 deletion HepG2 monoclonal cell lines by WB (n = 2). F) Nude mice were intraperitoneally inoculated with RNASET2^KO^ HuH-7 cells (n = 5) and tumors in the abdominal cavity were observed after 28 days. G) qRT-PCR analysis of *RNASET2* mRNA level in stably RNASET2^FLAG^ transfected MHCC97-H cells (n = 3). H) The levels of EMT-related proteins (N-cadherin, MMP9, MMP2, and Snail) in RNASET2 overexpressed MHCC97-H cells were determined by WB (n = 2). I-K) Functions of forced expression RNASET2 in the migration and invasion of MHCC97-H cells were identified by wound healing assay (I), trans-well migration assay (J), and trans-well invasion assay (K). The distance of the wound gaps was determined using ImageJ. The wound width was normalized against that at time 0 h. Quantification was shown below. Data represent three independent experiments in duplicate (n = 3). Bars for wound healing assays: 200 µm, for trans-well assays: 400 µm. L,M) Macroscopic views of lung metastasis (L) and lung weight (M) of mouse model *i.v.* injected with RNASET2^FLAG^ or empty vector (Vec) MHCC97-H cells (n = 5; *P* = 0.0018). N) Survival of lung metastatic mouse model triggered by RNASET2^FLAG^ and empty vector MHCC97-H cells (n = 10). O) H&E staining exhibited lung metastasis of RNASET2^FLAG^ MHCC97-H cells compared to controls in nude mice. The images in the below panel were shown with higher magnification (n = 5). Scale bars: 400 μm. Data are presented as mean ± SD. **p* < 0.05, ***p* < 0.01, ****p* < 0.001, *****p* < 0.0001 by two-tailed unpaired Student’s *t*-test (A,G and I-K and M) or log-rank (Mantel-Cox) test (N).


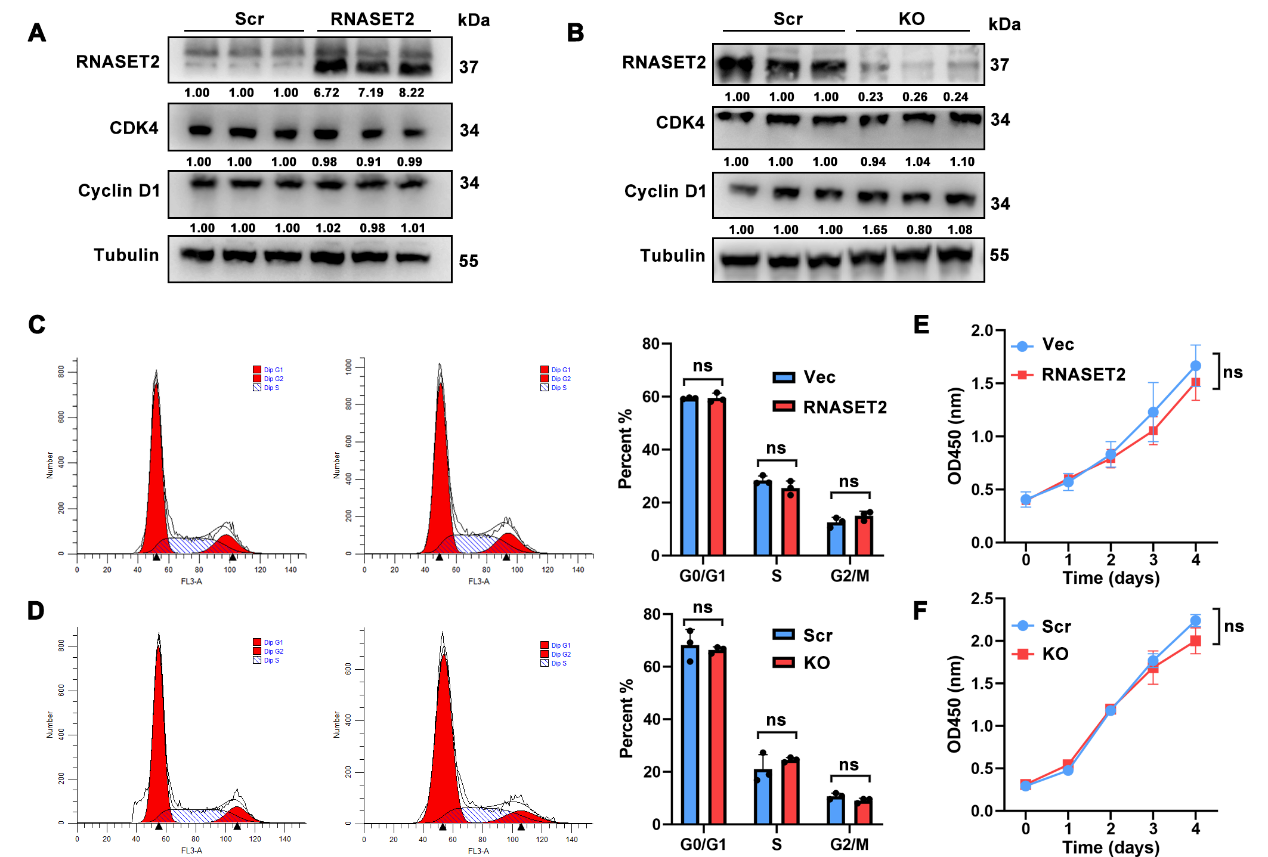


**Figure S2.** Effects of RNASET2 on HCC cells proliferation: A,B) The levels of CDK4 and Cyclin D1 in RNASET2-overexpressing MHCC97-H cells (A) and RNASET2^KO^ HuH-7 cells (B) were measured by WB (n = 3). C,D) Flow cytometry analysis of cell cycle in RNASET2-overexpressing MHCC97-H cells (C) and deficient HuH-7 cells (D). Quantification was shown right. Data represent three independent experiments in duplicate (n = 3). E,F) CCK8 assays were used to detect the cell viability of RNASET2-overexpressing MHCC97-H cells (E) and deficient HuH-7 cells (F) over 4 days (n = 3). Data are presented as mean ± SD. *ns* = not significant by two-tailed unpaired Student’s *t*-test (C,D), two-way ANOVA (E,F).

**
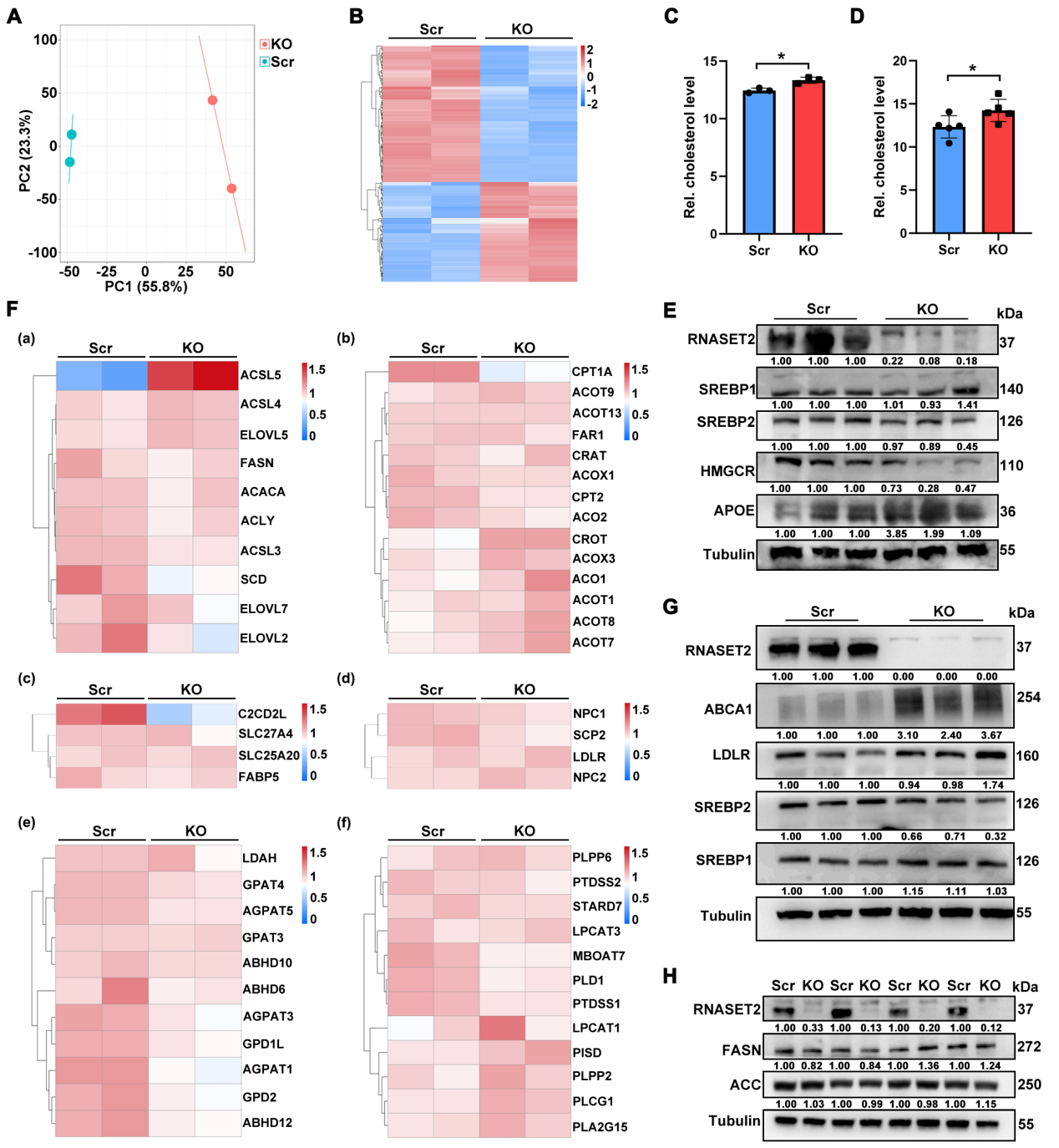
**

**Figure S3.** RNASET2 modulates lipid metabolism: A) Principal component analysis (PCA) of Scr- and RNASET2^KO^-Huh7 cells using proteomic assay (n = 2). B) Heatmap of all differentially expressed proteins in proteomic data. The protein levels were normalized as log2 (RNASET2^KO^ *versus* scrambled) (n = 2). C) The Amplex Red cholesterol assay kit was utilized to determine the cholesterol concentration in both control and RNASET2^KO^ HuH-7 cells (n = 3). D) The cholesterol assay kit was employed to assess the cholesterol levels in control and RNASET2^KO^ HuH-7 cells derived from induced lung tumor tissues (n = 5). E) WB assessed the levels of SREBP1, SREBP2, HMGCR, and APOE in RNASET2^KO^ and scrambled control HepG2 cells (n = 3). F) Heatmap of proteins associated with lipid metabolism, such as fatty acids synthesis (a), fatty acids oxidation (b), fatty acids transportation (c), cholesterol uptake (d), TAG metabolism (e), and phospholipid metabolism (f) in proteomic data (n = 2). G) Expression levels of ABCA1, LDLR, SREBP2, and SREBP1 in RNASET2 deletion and control HuH-7 cells were measured by WB (n = 3). H) FASN and ACC levels were evaluated using WB (n = 4). Data are presented as mean ± SD. **p* < 0.05 by two-tailed unpaired Student’s *t*-test (C, D).

**
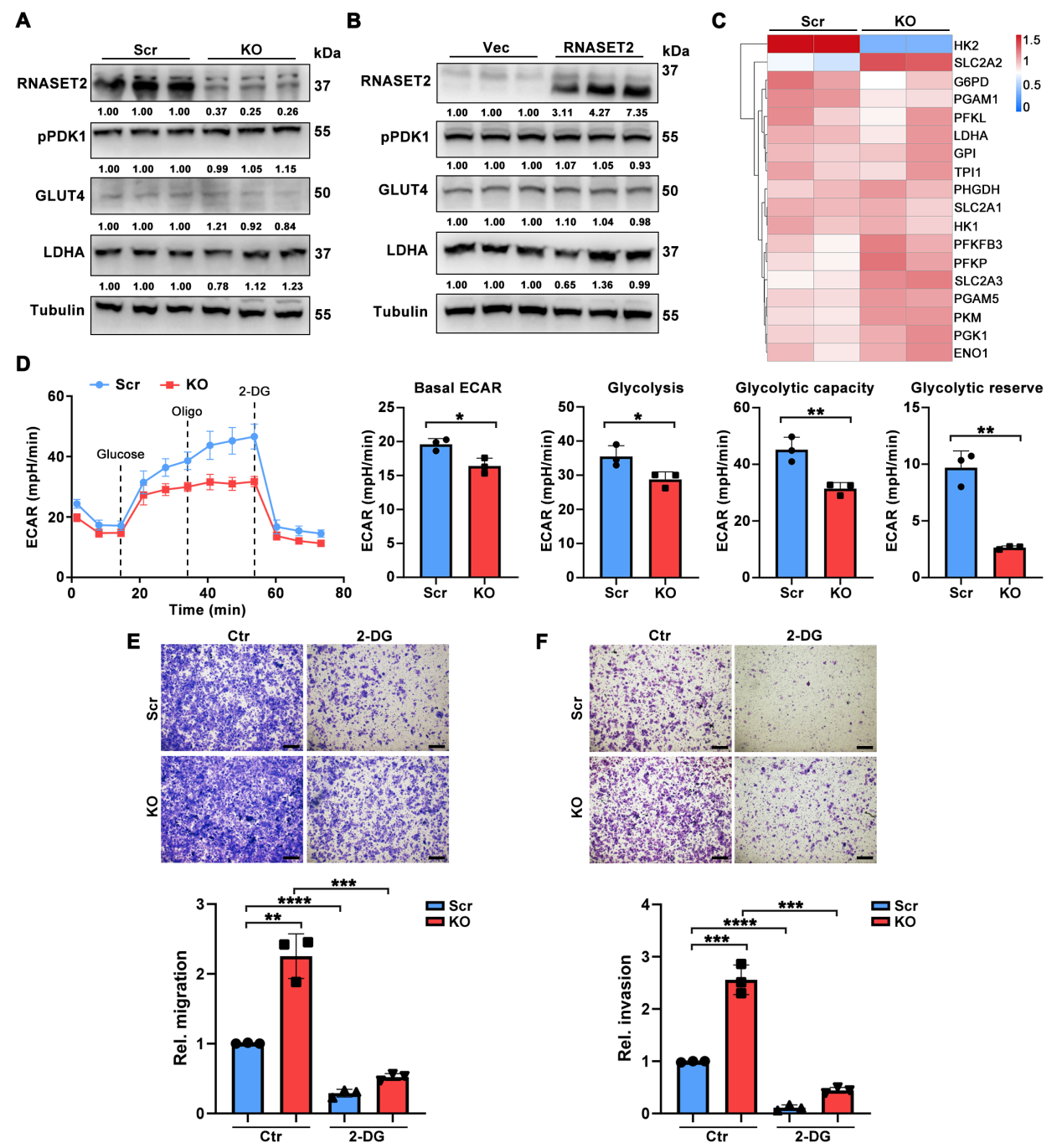
**

**Figure S4.** RNASET2 suppresses glycolysis of HCC cells: A,B) The levels of pPDK1, LDHA, and Glut4 in RNASET2^KO^ HuH-7 cells (A) and RNASET2-overexpresion MHCC97-H cells (B) were tested using WB (n = 3). C) Heatmap of enzymes associated with glycolysis metabolic pathway. D) Seahorse glycolysis stress test was conducted to measure the extracellular acidification rate (ECAR) of scrambled and RNASET2^KO^ HuH-7 cells (n = 3). E,F) The migration (E) and invasion (F) of scrambled- and RNASET2^KO^- HuH-7 cells in the absence or presence of 2-DG (10 mM) were determined by trans-well assays. Quantification was shown below (n = 3). Data are presented as mean ± SD. **p* < 0.05, ***p* <0.01, ****p* <0.001, *****p* <0.0001 by two-tailed unpaired Student’s *t*-test (D-F).

**
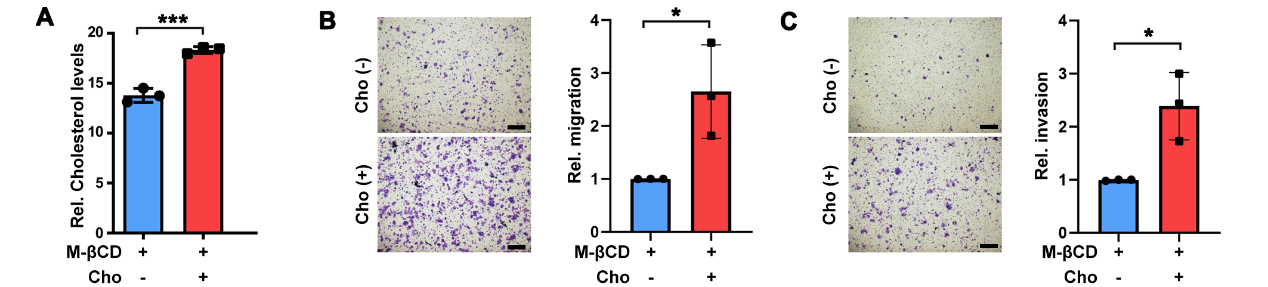
**

**Figure S5.** Cholesterol promotes metastasis of HCC cells: A) Amplex red cholesterol assay kit determination of cholesterol concentration in HuH-7 cells. The cells were cultured in medium with MβCD for 2 h followed by addition with exogenous cholesterol for another 2 h (n = 3). B,C) Roles of exogenous cholesterol in the migration and invasion of HuH-7 cells were determined by trans-well migration assay (B), and trans-well invasion assay (C) (n = 3). Quantification analysis was shown in the right panel. Bars for trans-well assays: 400 µm. Data are presented as mean ± SD. **p* < 0.05, ****p* <0.001 by two-tailed unpaired Student’s *t*-test (A-C).

**
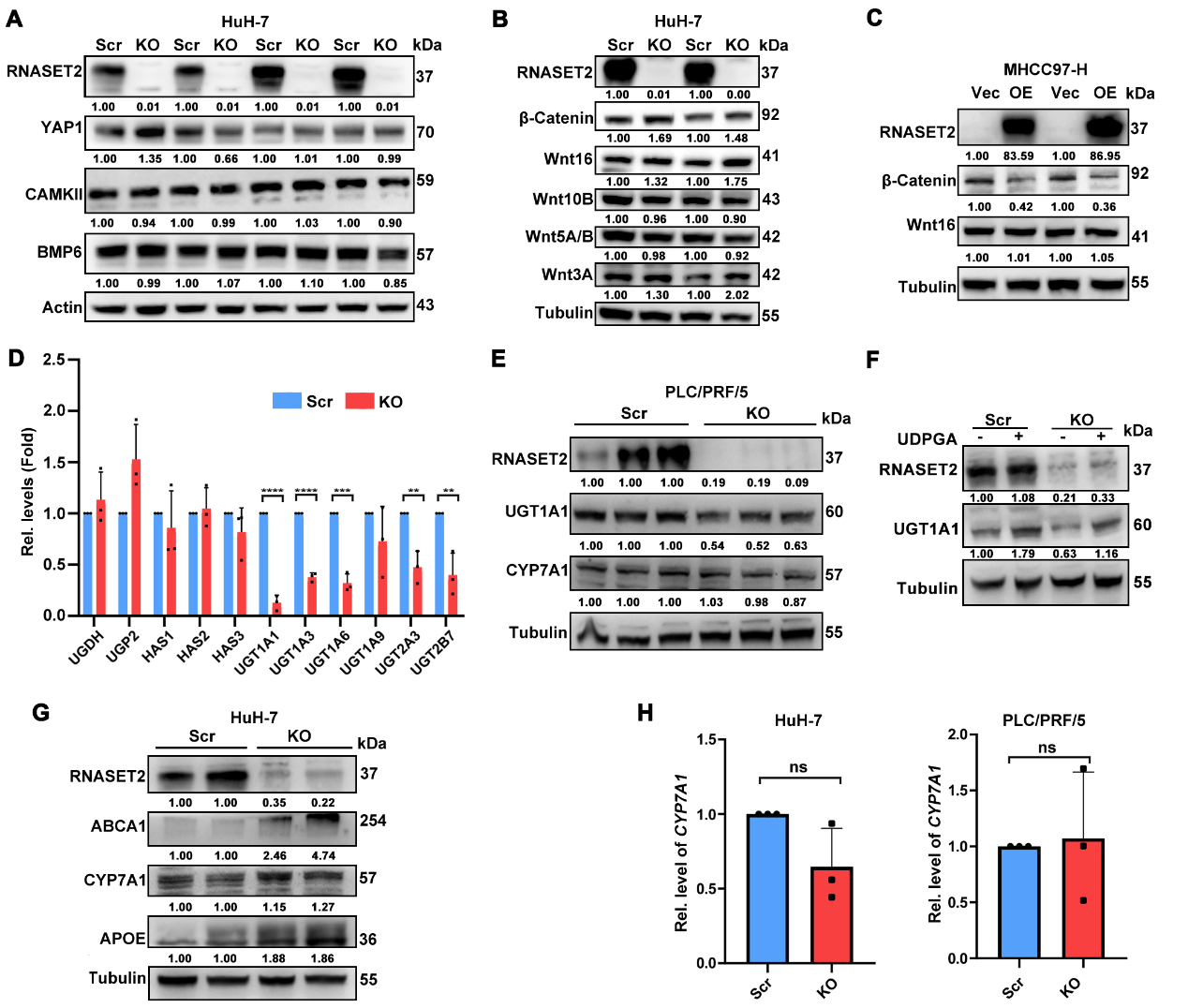
**

**Figure S6.** RNASET2 deletion blunts the level of UGT1A1 in HCC cells: A-C) Levels of CAMs, Hippo signaling pathway (A) and Wnt signaling pathway (B,C) associated proteins in RNASET2 elimination HCC cells were evaluated by WB (n = 4). D) qRT-PCR detected mRNA levels of enzymes involved in glucuronate metabolism of PLC/PRF/5^KO^ cells (n = 3). E) WB analysis of the levels of UGT1A1 and CYP7A1 in RNASET2^KO^ PLC/PRF/5 cells (n = 3). F) WB analysis of the protein level of UGT1A1 in control and RNASET2^KO^ HuH-7 cells with or without UDPGA (500 μM) treatment (n = 3). G) WB assessed the levels of ABCA1, CYP7A1, and APOE in RNASET2^KO^ HuH-7 cells (n = 4). H) mRNA levels of the limited enzyme CYP7A1 involved in bile acids biosynthesis (n = 3). Data are presented as mean ± SD. ***p* < 0.01, ****p* < 0.001, *****p* < 0.0001, *ns* = not significant by two-tailed unpaired Student’s *t*-test (D,H).

**
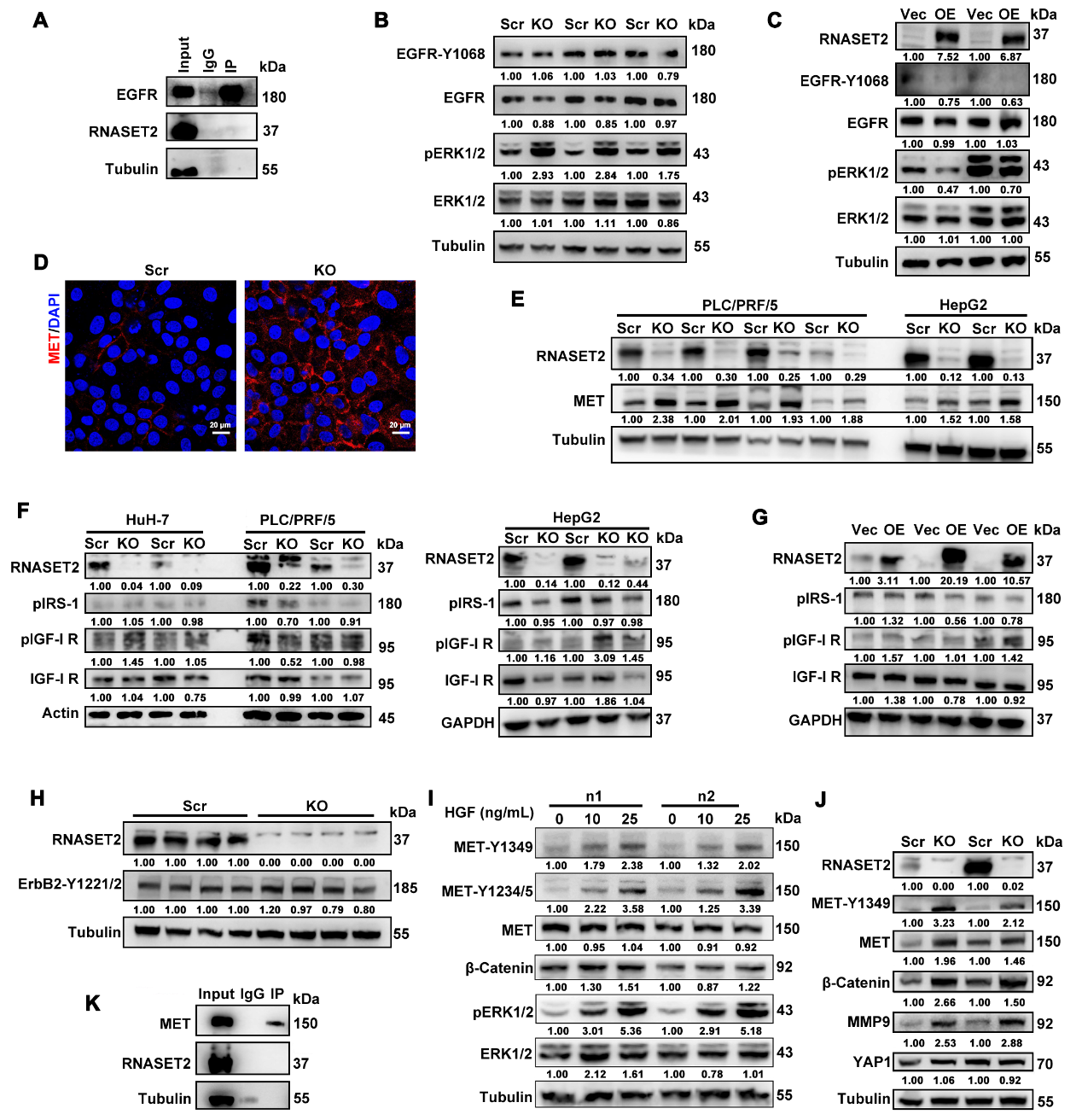
**

**Figure S7.** RNASET2 regulates RTKs signaling in HCC cells: A) Immunoprecipitation between EGFR and RNASET2 in MHCC97-H cells (n = 3). B,C) Effects of RNASET2 gain-or loss-of-function on the total and phosphorylated levels of EGFR and target proteins ERK1/2 in HuH-7 (B) and MHCC97-H (C) cells were assessed by WB (n = 3). D) Confocal microscopy of MET in scr- or RNASET2^KO^-PLC/PRF/5 cells (n = 3). Scale Bars: 20 μm. E) WB assays of MET production in scrambled- or RNASET2^KO^- PLC/PRF/5 and HepG2 cells (n = 2 to 4). F,G) WB examined the levels of IGF-I R, pIGF-I R or the substrate protein pIRS-1 in RNASET2 elimination HuH-7, PLC/PRF/5 and HepG2 cells (F) or RNASET2^OE^ MHCC97-H cells (G) (n = 2 to 3). H) WB detected the levels of ErbB2-Y1221/1222 in RNASET2^KO^ PLC/PRF/5 cells (n = 4). I) Serum starved PLC/PRF/5 cells followed by HGF treatment for 15 min were subjected to WB, the levels of β-catenin, phosphorylated MET, and phosphorylated ERK1/2 were determined (n = 4). J) WB measured the levels of total or phosphorylated MET, β-Catenin, MMP9, and YAP1 in RNASET2^KO^ HCC cells (n = 4). K) Immunoprecipitation of RNASET2^FLAG^ MHCC97-H cells with antibodies targeting MET or normal IgG (n = 3).

**
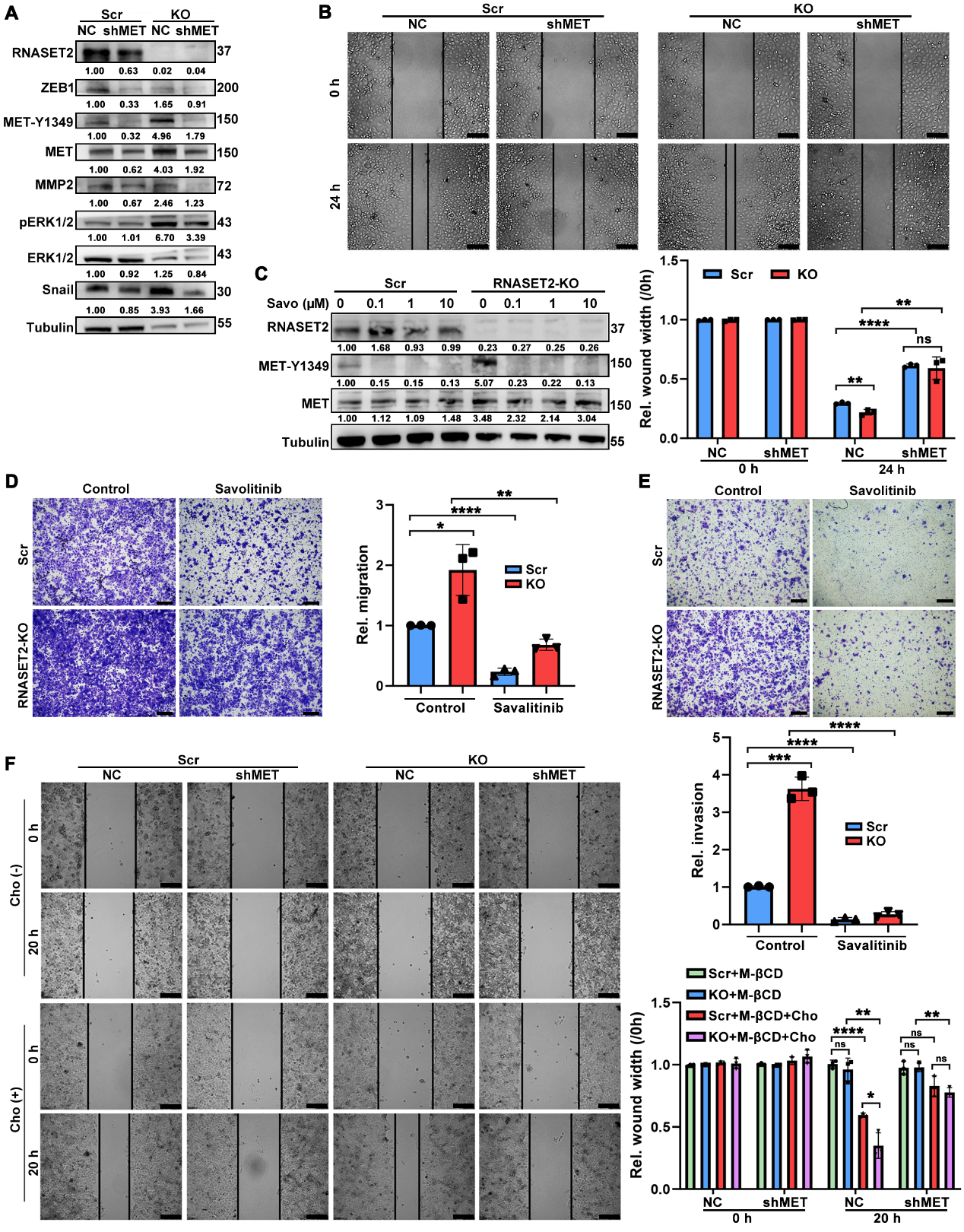
**

**Figure S8.** Inhibition of MET attenuates migration and invasion of RNASET2^KO^ HCC cells: A) Identification of the efficiency of *MET* knockdown and the levels of MET targeted proteins, including ZEB1, MMP2, Snail1, and phosphorylated ERK1/2, in RNASET2^KO^/shMET PLC/PRF/5 cells using WB (n = 3). B) Representative images of wound healing assays of RNASET2^KO^/shMET HuH-7 cells (n = 3). C) Analysis of the phosphorylation level of MET in RNASET2 ablation HuH-7 cells treated with indicated concentrations of savolitinib for 10 min by WB (n = 3). D,E) Representative images of migration (D) and invasion (E) assays of RNASET2^KO^ HuH-7 cells treated with 1 μM savolitinib for 48 h (n = 3). F) Wound healing assays of RNASET2^KO^ HuH-7 cells transfected with shMET in the presence or absence of exogenous cholesterol (n = 3). Quantification analysis were shown in the below (B,E) and right (D,F) panels. Scale bars for wound healing assays: 200 μm, for trans-well: 400 µm. Data are presented as mean ± SD. **p* < 0.05, ***p* <0.01, ****p* <0.001, *****p* <0.0001, *ns* = not significant by two-tailed unpaired Student’s *t*-test (B,D,E,F).


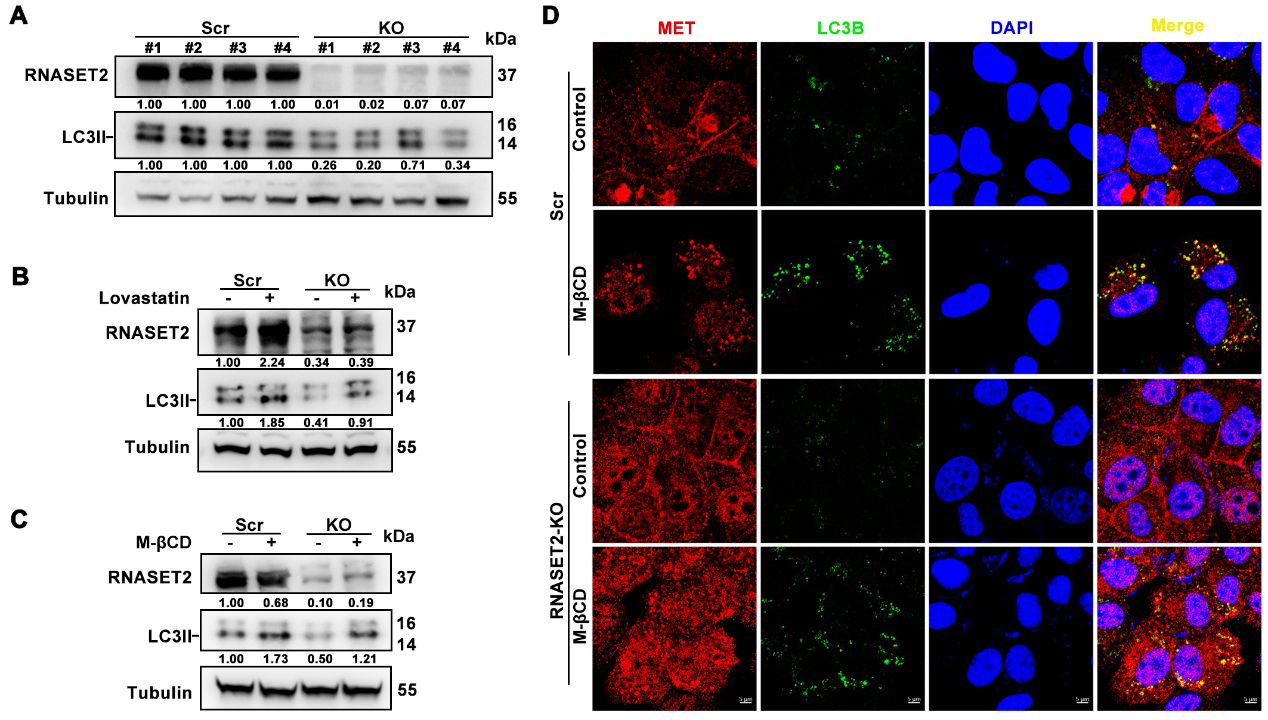


**Figure S9.** RNASET2 deficiency suppresses the autophagic degradation of MET through the accumulation of cholesterol: A) The level of LC3II in control and RNASET2^KO^ HuH-7 cells was assessed using Western blotting (n = 3). B) Western blot analysis of LC3II in control and RNASET2^KO^ HuH-7 cells, conducted in the absence or presence of 3 μM lovastatin (n = 3). C) Western blot analysis of LC3II levels in control and RNASET2^KO^ HuH-7 cells, treated with or without MβCD (0.75%) for 8 h (n = 3). D) Immunofluorescence staining of MET (red), LC3B (green), and DAPI (blue) in control and RNASET2^KO^ HuH-7 cells. The cells were pre-starved in a medium containing 20 μM hydroxychloroquine (HCQ) for 6 h and subsequently treated with or without MβCD (0.75%) for an additional 2 h (n = 3). Scale bars represent 5 μm.

**Table S1.** Clinical characteristics of participants.

| Participants No | Age | Sex | HCC | HBV (Pos/Neg) | Tumor Size (cm*cm*cm) | Cirrhosis | ELISA | IHC | WB |
| --- | --- | --- | --- | --- | --- | --- | --- | --- | --- |
| HCC-01 | 66 | M | Yes | Positive | 13*9*6 | Yes | √ | √ | √ |
| HCC-02 | 70 | M | Yes | Negative | 3.5*2.5*2.5 | No | √ | √ | √ |
| HCC-03 | 73 | M | Yes | Positive | 6*4*3.5 | No | √ | √ | √ |
| HCC-04 | 52 | M | Yes | Positive | 5*4.2*4 | Yes | √ | √ | √ |
| HCC-05 | 26 | M | Yes | Negative | 4.5*4.5*3.5 | No | √ | √ | √ |
| HCC-06 | 40 | M | Yes | Positive | 5.5*4*3.5 | Yes | √ | √ | √ |
| HCC-07 | 60 | M | Yes | Positive | 17*14*10 | No | √ | √ | √ |
| HCC-08 | 48 | M | Yes | Positive | 7*6*5 | No | √ | √ | √ |
| HCC-09 | 62 | M | Yes | Positive | 2.5*2*1.2 | No | √ | √ | √ |
| HCC-10 | 56 | M | Yes | Positive | 3*2.5*2 | Yes | √ | √ | √ |
| HCC-11 | 55 | M | Yes | Positive | 2.7*2.7*2.5 | Yes | √ | √ | NA |
| HCC-12 | 49 | M | Yes | Positive | 5.5*4.5*4.5 | Yes | √ | √ | NA |
| HCC-13 | 42 | M | Yes | Negative | 10*4*8 | No | √ | NA | NA |
| HCC-14 | 73 | M | Yes | Negative | 10.5*10*10 | No | √ | NA | NA |
| HCC-15 | 57 | M | Yes | Positive | 1.8*1.5*1.2 | Yes | √ | NA | NA |
| HCC-16 | 48 | M | Yes | Positive | 3*2*1 | Yes | √ | NA | NA |
| HCC-17 | 33 | M | Yes | Positive | 2*1.5*1.2 | Yes | √ | NA | NA |
| HCC-18 | 45 | M | Yes | Positive | 11*9.5*7 | No | √ | NA | NA |
| HCC-19 | 47 | M | Yes | Positive | 2*1.5*1.5 | Yes | √ | NA | NA |
| HCC-20 | 61 | M | Yes | Positive | 1.4*1.4*1 | Yes | √ | NA | NA |
| HCC-21 | 54 | M | Yes | Negative | 6.5*6*3.5 | No | √ | NA | NA |
| HCC-22 | 33 | M | Yes | Positive | 4*3*2.5 | Yes | √ | NA | NA |
| HCC-23 | 38 | M | Yes | Positive | 5*4.5*4 | Yes | √ | NA | NA |
| HCC-24 | 53 | M | Yes | Positive | 3*3*2.5 | Yes | √ | NA | NA |
| HCC-25 | 51 | M | Yes | Positive | 4*2*2 | No | √ | NA | NA |
| HCC-26 | 54 | M | Yes | Positive | 4.5*5*5 | No | √ | NA | NA |
| HCC-27 | 53 | M | Yes | Positive | 7*4*2 | No | √ | NA | NA |
| HCC-28 | 34 | M | Yes | Positive | 15*10*4 | No | √ | NA | NA |
| HCC-29 | 49 | M | Yes | Positive | 7*6*6 | No | √ | NA | NA |
| HCC-30 | 50 | M | Yes | Positive | 2.5*2*1.5 | Yes | √ | NA | NA |
| Donor-1 | 36 | M | No | Negative | No | No | √ | NA | NA |
| Donor-2 | 34 | M | No | Negative | No | No | √ | NA | NA |
| Donor-3 | 33 | M | No | Negative | No | No | √ | NA | NA |
| Donor-4 | 34 | M | No | Negative | No | No | √ | NA | NA |
| Donor-5 | 38 | M | No | Negative | No | No | √ | NA | NA |
| Donor-6 | 38 | M | No | Negative | No | No | √ | NA | NA |
| Donor-7 | 30 | M | No | Negative | No | No | √ | NA | NA |

(√ = assay performed; NA=assay not performed)

**Table S2.** qPCR primer sequences.

| Target gene | Primer forward | Primer reverse |
| --- | --- | --- |
| *RNASET2* | GGCATACTGGCCTGACGTAAT | CTTTTCCCACTCATGCTTCCAGA |
| *ACTIN* | ACCTTCTACAATGAGCTGCG | CCTGGATAGCAACGTACATGG |
| *UGDH* | CAACAGCGATTGGAATGGACC | TCTGGCAAATTCAGAGCCTCA |
| *UGP2* | AAGAAGTCATTCGGCAAGAGC | CCACCATTGAGTTTCACCACC |
| *UXS1* | AGCCTCCCCTCCAAACTACA | GGCCACTCGCACTTCCAC |
| *HAS1* | CTCGGAGATTCGGTGGACTA | GAAACTGCTGCAAGAGGTTATTC |
| *HAS2* | TCCTGGATCTCATTCCTCAGC | TGCACTGAACACACCCAAAATA |
| *HAS3* | GTGGCTCAACCAGCAAACC | CGTCAGCAGGAAGAGGAGAATG |
| *HYAL1* | GCCCTCTTCCTGACCTTACTC | CCTGGGTTGGCTACCACATC |
| *HYAL2* | CGCTGCCCTGATGTTGAG | CGGCTGTAGGTGGGTCGT |
| *UGT1A1* | CCAAAATCCACTATCCCAGGA | GGGTCATCGGGTGACCAA |
| *UGT1A3* | TTTCACCCTGACAACCTATGC | AGCTCCACACAAGACCTATGAT |
| *UGT1A6* | CTCCTTCGCTCATTTCAGAGAAT | CGGTCACTGAGAACCTCAACTAT |
| *UGT1A9* | CCCCCTTCCTCTATGTGTGTG | TCATACTCCGTAACAGGTGTTTG |
| *UGT2A3* | CGAAGCTGTGGGAAACTTCCA | GGGCCTTCCTAATGCCTTACT |
| *UGT2B7* | GATCCCAACAACTCATCCGCT | CAGCAGCTCACTACAGGGAA |
| *CYP7A1* | CCATAAGGTGTTGTGCCACGGAAA | GCCCAAATGCCTTCGCAGAAG |
